# Vaping leads tobacco consumption among university students in Arab countries: a study of behavioral and psychosocial factors associated with smoking

**DOI:** 10.3389/fpubh.2025.1636757

**Published:** 2025-08-06

**Authors:** Malik Sallam, Eman Khamis Alnazly, Adil Sajwani, Kholoud Al-Mahzoum, Yousef Alkhalaf, Aisha Aldaihani, Abdulrahman Aldousari, Mohammad Alhajeri, Meshari Almutairi, Shekha Alnajdi, Mohammad Alkhozam, Abdulaziz Muneer Alsubaiei, Naser Eisa, Sulaiman Altheyab, Doaa H. Abdelaziz, Noha O. Mansour, Mohammed Sallam

**Affiliations:** ^1^Department of Pathology, Microbiology and Forensic Medicine, School of Medicine, The University of Jordan, Amman, Jordan; ^2^Department of Clinical Laboratories and Forensic Medicine, Jordan University Hospital, Amman, Jordan; ^3^Department of Primary Care Nursing, Faculty of Nursing, Al-Ahliyya Amman University, Amman, Jordan; ^4^Hourani Center for Applied Scientific Research, Al-Ahliyya Amman University, Amman, Jordan; ^5^Department of Family Medicine, Mediclinic Parkview Hospital, Mediclinic Middle East, Dubai, United Arab Emirates; ^6^Department of Management, Mediclinic Parkview Hospital, Mediclinic Middle East, Dubai, United Arab Emirates; ^7^Sheikh Jaber Al-Ahmad Al-Sabah Hospital, Ministry of Health, Kuwait City, Kuwait; ^8^School of Medicine, The University of Jordan, Amman, Jordan; ^9^Department of Clinical Pharmacy, Faculty of Pharmacy, Al-Baha University, Al-Baha, Saudi Arabia; ^10^Department of Clinical Pharmacy, the National Hepatology and Tropical Medicine Research Institute, Cairo, Egypt; ^11^Department of Clinical Pharmacy and Pharmacy Practice, Faculty of Pharmacy, Mansoura University, Mansoura, Egypt; ^12^Department of Pharmacy, Mediclinic Parkview Hospital, Mediclinic Middle East, Dubai, United Arab Emirates; ^13^Department of Management, School of Business, International American University, Los Angeles, CA, United States; ^14^College of Medicine, Mohammed Bin Rashid University of Medicine and Health Sciences (MBRU), Dubai, United Arab Emirates

**Keywords:** nicotine vaping product, electronic nicotine delivery system, electronic cigarettes, health risk, health behavior, health policy, tobacco, vape

## Abstract

**Background:**

E-cigarettes use “vaping” is a growing public health concern. The Arabic-validated Vaping Attitude and Perceptions Scale (VAPeS) instrument assesses vaping determinants across five constructs: Social Influence, Perceived Harms, Vaping Pleasure, Behavioral Influences, and Economic and Self-Efficacy. We aimed to examine the prevalence of cigarette, e-cigarette, and narghile use among Arab university students and to identify the associations between demographic/VAPeS-related variables and the smoking attitudes among vapers.

**Methods:**

A cross-sectional survey was distributed online among university students across Arab countries during January–April 2025. Prevalence estimates were calculated using one-sample proportions with Wilson Score confidence intervals. The modified VAPeS scale comprising four constructs after confirmatory factor analysis (Social Influence, Perceived Benefits, Behavioral Influence - Risk, and Behavioral Influence - Situational Triggers) was used to assess tobacco use attitudes. The primary outcome was the Endorsement of Tobacco Use Attitude Score (ETUAS). Multivariate analyses using multinomial logistic and linear regressions were conducted to examine factors associated with tobacco use attitudes.

**Results:**

Among 1,338 university students surveyed, the majority were from Kuwait (21.0%), Egypt (18.5%), Kingdom of Saudi Arabia (KSA, 17.8%), Jordan (16.7%), and the United Arab Emirates (UAE, 10.0%). Vaping was the most prevalent form of tobacco use (21.2%), surpassing narghile (12.9%) and cigarette smoking (10.8%). Multivariate analyses revealed that male students were more likely to engage in all three tobacco use forms. Male sex was associated with higher prevalence of vaping (adjusted odds ratio (AOR) = 6.97; *p* < 0.001), with higher odds among UAE students (AOR = 2.31; *p* = 0.013), and lower odds among those studying in Egypt, Jordan, and KSA. Among current smokers, the mean ETUAS indicated a moderate level of agreement with attitudinal statements endorsing tobacco use (3.25 ± 0.92). In linear regression among vapers, male sex (*B* = −0.325; *p* = 0.003), Social Influence (*B* = 0.300; *p* < 0.001), and Behavioral Influence - Situational Trigger (*B* = 0.205; *p* = 0.002) were significantly associated with favorable attitudes toward tobacco use.

**Conclusion:**

This multinational study found vaping to be the leading form of tobacco use among Arab university students. Favorable attitudes toward tobacco use were associated with male sex, social influence, and the situational triggers. Interventions should target social normalization, strengthen regulations, and apply tools like VAPeS to understand youth vaping risks.

## 1 Introduction

Few public health threats have lasted as persistently as tobacco use (1). Once considered a symbol of leisure and prestige (2), tobacco use has been well-recognized as a leading cause of chronic illnesses, premature mortality, and staggering economic burden (3). Specifically, tobacco use is now unquestionably linked to a wide range of debilitating and life-threatening conditions, including but not limited to lung cancer, chronic obstructive pulmonary disease (COPD), coronary artery disease, stroke, reproductive dysfunction, ocular degeneration, and immune dysregulation (4–10). Despite the reported global declines in smoking prevalence since 1990 according to the Global Burden of Disease Study 2019, tobacco use remains a major global health challenge (11). Tobacco use was estimated to account for 7.69 million deaths and 200 million disability-adjusted life-years in 2019, with rising absolute numbers of smokers due to population growth (11).

Tobacco use can be defined as the consumption of products derived from the tobacco plant, including combustible forms such as cigarettes, cigars, and waterpipes (narghile), as well as non-combustible alternatives like chewing tobacco, snuff, and, more recently, electronic nicotine delivery systems (e.g., e-cigarettes and vaping devices) (12–14). These products vary in mode of nicotine delivery and cultural adoption and use, but all would result in exposure to harmful substances, including nicotine, carcinogens, and toxicants that contribute to a wide spectrum of chronic diseases (13, 15, 16).

Despite decades of concerted efforts by the global medical and public health communities to restrain tobacco consumption, it remains a persistent and complex challenge (17, 18). Effective interventions to reduce tobacco use include public anti-smoking campaigns, smoking bans, graphic health warnings on packaging, and increased taxation on tobacco products (19). However, tobacco use with its related giant industry remained highly adaptable, deeply embedded in cultural practices, and resistant to uniform policy interventions (17, 20, 21).

This tobacco epidemic is particularly pronounced in the Arab world, with several countries ranking among those with the highest tobacco use rates globally. Specifically, the Middle East and North Africa (MENA) region have witnessed the largest relative increase in the number of smokers since 1990—a staggering 104.1% rise among both males and females as shown by (11). According to the World Health Organization (WHO) 2025 projections for age-standardized estimates of current tobacco use, several Arab countries are expected to maintain alarmingly high tobacco use prevalence rates (22). Jordan leads the region with an estimated 36.3% of the population currently using tobacco, followed by Lebanon (34.1%) and Egypt (25.8%) (22). The governmental and religious institutions across the Arab region have issued formal regulations and religious decrees discouraging tobacco use (23). Nevertheless, the widespread availability and social normalization of smoking—especially through culturally ingrained practices such as narghile use—continue to sustain high levels of tobacco consumption in Arab countries (24–27).

Alarmingly, tobacco use remains prevalent among Arab youth, including those in higher education settings where health literacy is presumed to be higher (26, 28–31). This persistence reflects a complex interplay of cultural norms, social identity, and academic stress (32, 33). Within this context, the use of electronic cigarettes (e-cigarettes; vaping) have emerged as a paradoxical product (34, 35). Though originally designed as a harm-reduction tool to support smoking cessation (36, 37), e-cigarettes have evolved into lifestyle commodities characterized by attractive designs, appealing flavors, and aggressive digital marketing (21, 38–41). Nowadays, vaping is increasingly adopted not as a cessation aid but as a fashionable, socially accepted practice, thereby complicating public health strategies aimed at reducing nicotine dependence (42–45). Importantly, emerging evidence further complicates the perceived safety of vaping, as it has been shown to be associated with asthma, bronchitis, emphysema, COPD, various cardiovascular conditions, periodontal disease, and suicidal behavior particularly among adolescents (46–50).

Similar to conventional tobacco use, the adoption of vaping among young adults is not shaped solely by neurobiological addiction pathways but by psychosocial perceptions—how it is viewed and socially endorsed (51–53). Among Arab university students, including those in health-related programs, vaping has been perceived as a cleaner, less harmful alternative to cigarette smoking and, in some cases, as a tool to help in smoking cessation (54–59). This favorable perception of vaping persists despite growing scientific evidence associating e-cigarette use with endothelial dysfunction, pulmonary inflammation, and cardiovascular risk (60–64). The primary public health concern surrounding vaping stems from uncertainties regarding its long-term health effects (65–67). Unlike conventional tobacco products such as cigarettes with well-established health risks that are widely publicized—vaping occupies a regulatory and perceptual gray zone (68). This ambiguity increases vaping’s appeal among youth, who often perceive it as a more discreet and socially acceptable alternative to traditional cigarette smoking (43, 69).

To address the determinants of vaping, a behavioral science perspective is important—particularly through the framework of the Theory of Planned Behavior (TPB), which emphasizes that health-related behaviors are shaped by individual attitudes, perceived social norms, and perceived behavioral control (70, 71). While TPB has been extensively applied across public health domains, its targeted application to vaping behavior is still explored to offer a novel and valuable framework for understanding the psychosocial drivers of e-cigarette use (72–74). The recent development of the Vaping Attitudes and Perceptions Scale (VAPeS), tailored for university student populations, provides a robust operationalization of the TPB (75). The VAPeS instrument translates the core TPB constructs into five distinct and measurable dimensions: Social Influence, Harm Perception, Perceived Vaping Pleasure, Behavioral Influence, and Economic Factors and Self-Efficacy (75). These dimensions offer comprehensive insights of vaping determinants among youth and hold particular utility for public health initiatives targeting the growing prevalence of e-cigarette use in Arab countries.

Thus, the current study aimed to employ the VAPeS instrument across multiple Arab countries to investigate vaping-related behaviors and perceptions among university students. Specifically, the study objectives were: (1) to estimate the prevalence of cigarette smoking, vaping, and narghile use among university students in Arab countries; (2) to assess students’ attitudes toward tobacco use as a primary outcome; and (3) to examine how demographic characteristics and VAPeS constructs contribute to shaping tobacco use attitudes among the students who use vaping products.

## 2 Methods

### 2.1 Study design

This cross-sectional study aimed to assess the prevalence of tobacco use among university students in Arab countries and the determinants of attitude to tobacco use via the validated VAPeS scale (75). Data were collected via an electronically self-administered questionnaire to facilitate broad and rapid distribution across geographically diverse settings (76). A convenience sampling approach was employed to enhance participation within the target population, acknowledging the exploratory nature of the study and the logistical challenges of establishing a unified sampling frame across countries (77). In addition, a snowball sampling technique was employed as a supplementary outreach method, whereby participants were encouraged to share the survey within their university and social networks. This dual-sampling approach was applied uniformly across all participating countries to maximize reach and participation given the absence of a unified sampling frame.

Eligible participants were individuals aged 18 years or older, currently enrolled in a university within an Arab country, and possessing sufficient proficiency in Arabic to complete the survey instrument. Participation was entirely voluntary, with strict assurances of anonymity and confidentiality. Respondents who did not provide informed consent or submitted incomplete survey responses were excluded from the final analysis. The study protocol received ethical approval from the Institutional Review Board (IRB) at the Deanship of Scientific Research at Al-Ahliyya Amman University.

### 2.2 Survey distribution

To enhance outreach, a convenience sampling strategy was employed as the primary recruitment method, supplemented by snowball sampling, whereby initial participants were encouraged to disseminate the survey link within their personal university students’ networks (78). Data were collected over a 101-day period (January 6 to April 17, 2025) using an online, self-administered questionnaire hosted on SurveyMonkey (SurveyMonkey Inc., San Mateo, California, USA). The survey was available in Arabic and distributed without incentives for participation.

Recruitment was facilitated via the widely used social media platforms in Arab countries including Twitter (X), LinkedIn, WhatsApp, Facebook Messenger, and Telegram. Ten co-authors, Kuwaiti medical students studying in Jordan, and other coauthors based in universities and health institutions across Jordan, Egypt, Kingdom of Saudi Arabia (KSA), and the United Arab Emirates (UAE), played a key role in expanding the survey’s geographic reach.

Quality control (QC) measures were implemented to ensure data integrity. These QC measures included restriction of the survey to one response per IP address and inclusion of items designed to detect internally contradictory answers. Upon accessing the survey, all participants were required to read a standardized information sheet and provide electronic informed consent with “Yes” answer being mandatory for inclusion. The questionnaire assessed demographic characteristics, patterns and frequency of tobacco use, attitudes toward tobacco use, and its psychosocial determinants measured via the validated VAPeS instrument (75). The full questionnaire is available in Supplementary material.

### 2.3 Sample size calculation

To ensure the reliability of country-level prevalence estimates of tobacco use in the absence of data *a priori*, a minimum sample size of 97 participants per country was required. This threshold was determined using the Epitools—sample size calculations, which is an online tool for estimating a single proportion under pre-specified conditions (79). Assuming an unknown prevalence, a conservative estimate of 50% was applied with a precision of ±5% and a 95% confidence level (79, 80).

### 2.4 Collection of demographic characteristics and Tobacco use history

Participants were required to provided self-reported demographic information, including age (scale variable later categorized into three categories: 18–20 years vs. 21–24 years vs. >24 years), sex (male vs. female), nationality (Arab country of origin), current country of university location, type of university (public vs. private), academic faculty (categorized as health vs. scientific vs. humanities), self-reported monthly household income (low vs. moderate vs. high), and monthly personal allowance (low vs. moderate vs. high).

Tobacco use history was assessed through a series of items capturing lifetime and current usage of various tobacco products. Participants were asked whether they had ever used any tobacco product (yes/no) and to report their current tobacco use status (non-user, ex-user, or current user). Specific questions addressed current cigarette smoking, e-cigarette (vaping) use, and narghile (waterpipe) use, including binary responses (yes/no) and the use frequency. Cigarette smoking frequency was categorized into four groups: 1–5 cigarettes per day, 6–10 per day, 11–20 per day, and more than 20 per day. Vaping frequency was classified as: once weekly or less, multiple times per week but not daily, daily with fewer than 10 sessions, and daily with 10 or more sessions. Narghile (waterpipe) use was categorized as: once weekly or less, multiple times per week but not daily, and daily use.

To ensure data quality, internal QC checks were embedded within the survey to identify contradictory or careless responses. Examples of such inconsistencies included: reporting never using tobacco while indicating current cigarette, vape, or narghile use; identifying as a non-smoker while specifying a daily cigarette consumption rate; or stating current use of a tobacco product while denying any frequency of use. Cases with inconsistencies in self-reported tobacco behavior were flagged for exclusion from the final analysis.

### 2.5 Survey instrument

The primary outcome, Endorsement of Tobacco Use Attitude Score (ETUAS), was evaluated using a 4-item scale administered exclusively to participants who indicated current tobacco product(s) use. These items were selected based on the WHO Global Adult Tobacco Survey (81). Each item was rated on a 5-point Likert scale (1 = strongly disagree to 5 = strongly agree). The items were: (1) I enjoy the experience of smoking and have no plans to stop; (2) I feel smoking is a necessary part of my daily routine; (3) I feel it would be difficult for me to quit smoking; and (4) I plan to continue smoking even though I know of its health risks. The ETUAS was calculated as the mean score of these four items, with higher scores indicating favorable attitude and endorsement of tobacco use. The ETUAS demonstrated fairly high internal consistency as demonstrated by a Cronbach’s *α* value of 0.783 (82).

To assess secondary psycho-social and economic constructs that could influence tobacco use attitudes, the previously Arabic-validated VAPeS scale was administered to participants who reported current vape use (75). The scale comprises five conceptual constructs: (1) Social Influence; (2) Harm Perception; (3) Vaping Pleasure; (4) Behavioral Influences; and (5) Economic and Self-Efficacy Factors (75). Each item was rated on a 5-point Likert scale ranging from 1 (strongly disagree) to 5 (strongly agree). Construct-specific scores were calculated by summing item responses within each construct and dividing by the number of items, yielding mean construct scores where higher values indicated stronger agreement or disagreement depending on the application of reverse coding of the items that conveyed anti-vaping attitude.

Exploratory factor analysis (EFA) of 21 items was conducted using IBM SPSS Statistics for Windows, Version 26.0. Armonk, NY: IBM Corp, with oblimin rotation, yielding five components explaining 62.4% of the variance. Five anti-vaping items were reverse-coded to ensure consistent directionality. One item was removed for low loading. Confirmatory factor analysis (CFA) using maximum likelihood in JASP software (Version 0.19.0) (83), confirmed a four-factor scale herein termed modified VAPeS scale (m-VAPeS) comprising: “Social Influence,” “Perceived Benefits,” “Behavioral Influence - Risk,” and “Behavioral Influence - Situational Triggers.” Items with loadings <0.50 and sub-scales with Cronbach’s *α* < 0.70 were excluded, including “Self-Efficacy.” Model fit was acceptable (Comparative Fit Index (CFI) = 0.938, Root mean square error of approximation (RMSEA) = 0.074, Standardized root mean square residual (SRMR) = 0.054), and internal consistency was strong (α = 0.771–0.799) with a total internal consistency of the m-VAPeS of 0.763 (Supplementary material).

The Social Influence construct included four items that measure peer pressure, perceived social acceptance, and media portrayal of vaping. The items were: (1) I feel pressured to smoke by my peers; (2) My role models in life consume tobacco in any form; (3) The portrayal of vaping in movies and series influences how I perceive it; and (4) I believe that vaping helps with social acceptance. The Social Influence construct showed fairly high internal consistency as demonstrated by a Cronbach’s *α* value of 0.787 (82).

The Perceived Benefits construct consisted of three items: (1) I believe that vaping is less harmful compared to cigarettes; (2) I believe that vaping is less harmful compared to narghile; and (3) Vaping can help smokers quit or reduce smoking. The Perceived Benefits construct showed fairly high internal consistency as demonstrated by a Cronbach’s *α* value of 0.776 (82).

The Behavioral Influence - Risk construct comprised two items: (1) I believe that vaping is associated with health risks; and (2) I believe that vaping can lead to nicotine addiction; with a Cronbach’s *α* value of 0.799. Finally, the Behavioral Influence - Situational Triggers construct comprised three items: (1) Academic stress affects my vaping habits; (2) I believe that vaping helps in stress reduction; and (3) I can easily obtain e-cigarettes with Cronbach’s α value of 0.771.

### 2.6 Statistical and data analysis

The statistical analysis was conducted using IBM SPSS Statistics for Windows, Version 26.0. Armonk, NY: IBM Corp. Descriptive statistics were used to summarize the study variables, including measures of central tendency (mean) and dispersion (standard deviation, SD). The prevalence of current cigarette smoking, vaping (e-cigarette use), and narghile (waterpipe) use was estimated using one-sample proportion tests with 95% confidence intervals (CIs) calculated via the Wilson Score method, which provides accurate estimates (84). All proportions were based on the total number of valid respondents (*N* = 1,338).

Univariate analyses were performed using the chi-squared (χ^2^) test for associations between categorical variables. For associations between categorical variables and continuous (scale) variables—specifically the VAPeS sub-scale scores and the ETUAS—non-parametric tests were employed, including the Mann–Whitney *U* (M-W) test and Kruskal–Wallis H (K-W) test, due to non-normality of the continuous variables as confirmed by the Kolmogorov–Smirnov test for normality (*p* < 0.001 for all scale variables).

Multivariate analyses were conducted for variables with a *p* value <0.100 in univariate analysis to reduce the risk of multivariate model overfitting (85). Multinomial logistic regression was used to assess adjusted odds ratios (AORs) and 95% CIs for categorical outcomes, while linear regression was used for continuous outcome variables. Multicollinearity was assessed using the Variance Inflation Factor (VIF) with cut-off set at VIF > 3 (86). A *p* value <0.050 was considered statistically significant.

For analytic purposes, nationalities were classified into six groups: Egypt, Jordan, KSA, Kuwait, UAE, and an “Other” category. The latter comprised respondents from multiple countries (e.g., Syria, Iraq, Palestine, Lebanon, Sudan, Tunisia, Libya, Yemen, Bahrain, and Morocco) that individually did not reach the sample size threshold for separate analysis. This grouping was retained to preserve the regional breadth of the study while acknowledging its analytical heterogeneity.

EFA was conducted using IBM SPSS Statistics for Windows, Version 26.0. Armonk, NY: IBM Corp with principal component analysis (PCA) extraction and oblimin rotation. Sampling adequacy was assessed via the Kaiser-Meyer-Olkin (KMO) measure and Bartlett’s Test of Sphericity. Items with low loadings or conceptual misfit were excluded. CFA was performed in JASP software (Version 0.19.0) using maximum likelihood (ML) estimation Jasp (83). Model fit was evaluated using standard indices: χ^2^ test, Comparative Fit Index (CFI), Tucker-Lewis Index (TLI), RMSEA with 90% CI and *p* value, and SRMR. Constructs with Cronbach’s *α* < 0.70 or item loadings < 0.50 were excluded to ensure internal consistency and convergent validity.

## 3 Results

### 3.1 Description of the study sample and QC measures

Of the 1973 students who accessed the survey, 36 (1.8%) did not provide informed consent and were excluded. An additional 315 participants (16.0%) did not complete the questionnaire and were excluded from the final analysis, yielding a final sample of 1,622 respondents. The QC check was conducted to assess the internal consistency of responses related to tobacco use. Several discrepancies were identified as follows. Seven participants reported never using any tobacco products but simultaneously identified as current smokers. Conversely, 30 participants reported prior tobacco use yet claimed to have never smoked.

Inconsistencies also emerged between smoking status and product-specific use: three participants indicated they were not current smokers yet reported current cigarette use; 25 identified as ex-smokers but reported current vaping use; and three participants who stated they had never smoked indicated current vaping behavior. Similarly, 26 participants who described themselves as ex-smokers reported current narghile use, and five participants who reported never smoking identified as current narghile users. Additional inconsistencies were found in the reported frequency of use. Eighteen participants identified as non-smokers while providing a positive count for cigarettes smoked daily. Conversely, 31 participants self-identified as current smokers but reported “I do not smoke cigarettes currently” when asked about frequency of use. Among vape users, three participants denied current use yet reported using vape multiple times per day; 11 participants denied ever using vape but reported current use; and three current vape users stated that they had never vaped in the frequency item. Nineteen participants denied current narghile use while indicating narghile use frequency, and one participant reported current narghile use while selecting “I do not use narghile” in the frequency question. Additionally, 99 self-identified ex-smokers proceeded to answer attitude questions specific to current smoking, suggesting potential misinterpretation of survey logic or misclassification. Following QC check, the final sample comprised 1,338 participants as shown in Table 1.

**Table 1:** General features of the study sample (*N* = 1,338).

Variable	Category	Count	Percentage
Age category	18–20 years	475	35.5%
	21–24 years	665	49.7%
	>24 years	198	14.8%
Sex	Male	566	42.3%
	Female	772	57.7%
Nationality	Egypt	248	18.5%
	Jordan	224	16.7%
	Kuwait	281	21.0%
	KSA^c^	238	17.8%
	UAE^d^	134	10.0%
	Other^e^	213	15.9%
University location	Egypt	246	18.4%
	Jordan	361	27.0%
	Kuwait	117	8.7%
	KSA	245	18.3%
	UAE	237	17.7%
	Other^f^	132	9.9%
University type	Public	1,011	75.6%
	Private	327	24.4%
Faculty	Health	950	71.0%
	Scientific	233	17.4%
	Humanities	155	11.6%
Monthly family income^a^	Low	89	6.7%
	Moderate	1,000	74.7%
	High	249	18.6%
Monthly allowance^b^	Low	165	12.3%
	Moderate	949	70.9%
	High	224	16.7%

a Monthly family income: Self-reported by the participant.

b Monthly allowance: Self-reported by the participant.

c KSA: Kingdom of Saudi Arabia.

d UAE: United Arab Emirates.

e Other: Includes participants from Syria, Iraq, Oman, Palestine, Bahrain, Lebanon, Morocco, Qatar, Yemen, Sudan, Algeria, Tunisia, Libya, and other unspecified countries.

^f^ Other: Includes participants studying at universities located in Bahrain, Oman, Qatar, Morocco, Lebanon, Syria, Yemen, Tunisia, Algeria, Iraq, Palestine, Sudan, Libya, and other unspecified countries.

Most participating student were aged 21–24 years (49.7%), with females comprising a slight majority (57.7%). Five nationalities met the minimum sample size threshold required for country-level analysis: Kuwait (*n* = 281, 21.0%), Egypt (*n* = 248, 18.5%), KSA (*n* = 238, 17.8%), Jordan (*n* = 224, 16.7%), and the UAE (*n* = 134, 10.0%). The majority were enrolled in universities located in Jordan (27.0%), Egypt (18.4%), or KSA (18.3%), with 75.6% attending public universities. Most participants were enrolled in health-related faculties (71.0%). Regarding socio-economic indicators, 74.7% reported moderate monthly family income, and 70.9% reported moderate monthly personal allowance (Table 1).

### 3.2 Prevalence and factors associated with tobacco use in the study sample

Among the 1,338 university students included in final analysis, vaping was the most commonly reported form of tobacco use, with a prevalence of 21.2% (95% CI: 19.1–23.5%), followed by narghile use at 12.9% (95% CI: 11.2–14.8%) and cigarette smoking at 10.8% (95% CI: 9.3–12.6%), as estimated using Wilson Score CIs. A total of 45 participants (3.4%) reported using all three forms of tobacco—cigarettes, vaping, and narghile—concurrently. Dual use was also common: 86 participants (6.4%) reported using both cigarettes and vaping, 86 (6.4%) reported using both vaping and narghile, and 14 (1.0%) reported using both cigarettes and narghile.

**Figure 1:**
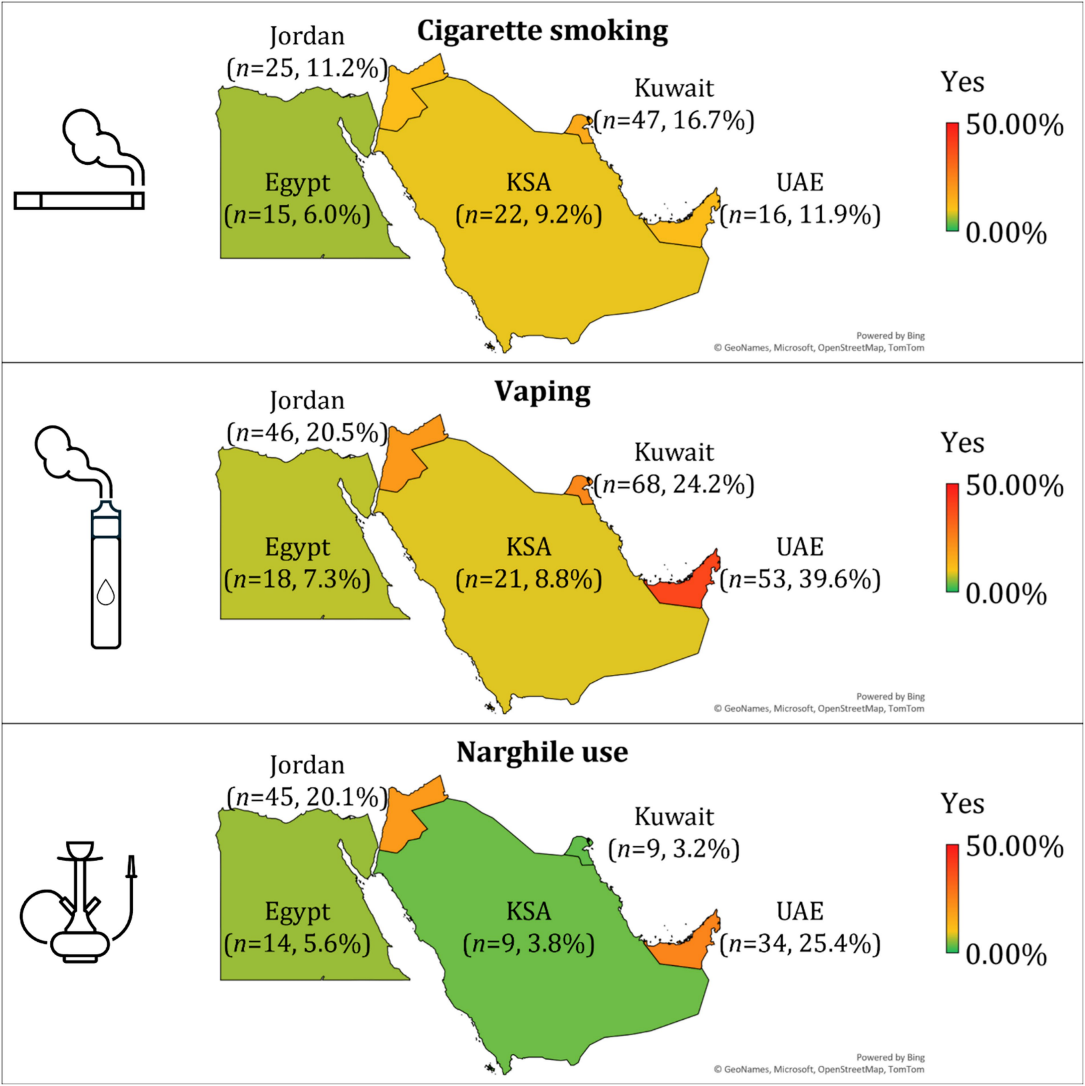
Prevalence of cigarette smoking, vaping, and narghile use among university students in five Arab countries. The number of participants (n) and the corresponding prevalence percentage are indicated within each country, based on the subset of respondents who reported tobacco use. Color gradients reflect prevalence levels, with red indicating higher rates and green indicating lower rates. KSA: the Kingdom of Saudi Arabia; UAE: the United Arab Emirates. The map was generated in Microsoft Excel, powered by Bing, © GeoNames, Microsoft, OpenStreetMap, TomTom, Wikipedia. We are neutral with regard to jurisdictional claims in this map. The symbols were generated in Microsoft PowerPoint.

Among university students in five Arab countries, vaping emerged as the most prevalent form of tobacco use in the UAE (39.6%), followed by Kuwait (24.2%) and Jordan (20.5%). Cigarette smoking was the highest in Kuwait (16.7%) and Jordan (11.2%), whereas narghile use was most commonly reported in the UAE (25.4%) and Jordan (20.1%). In contrast, Egypt and KSA reported consistently lower prevalence across all three tobacco products (Figure 1).

**Figure 2:**
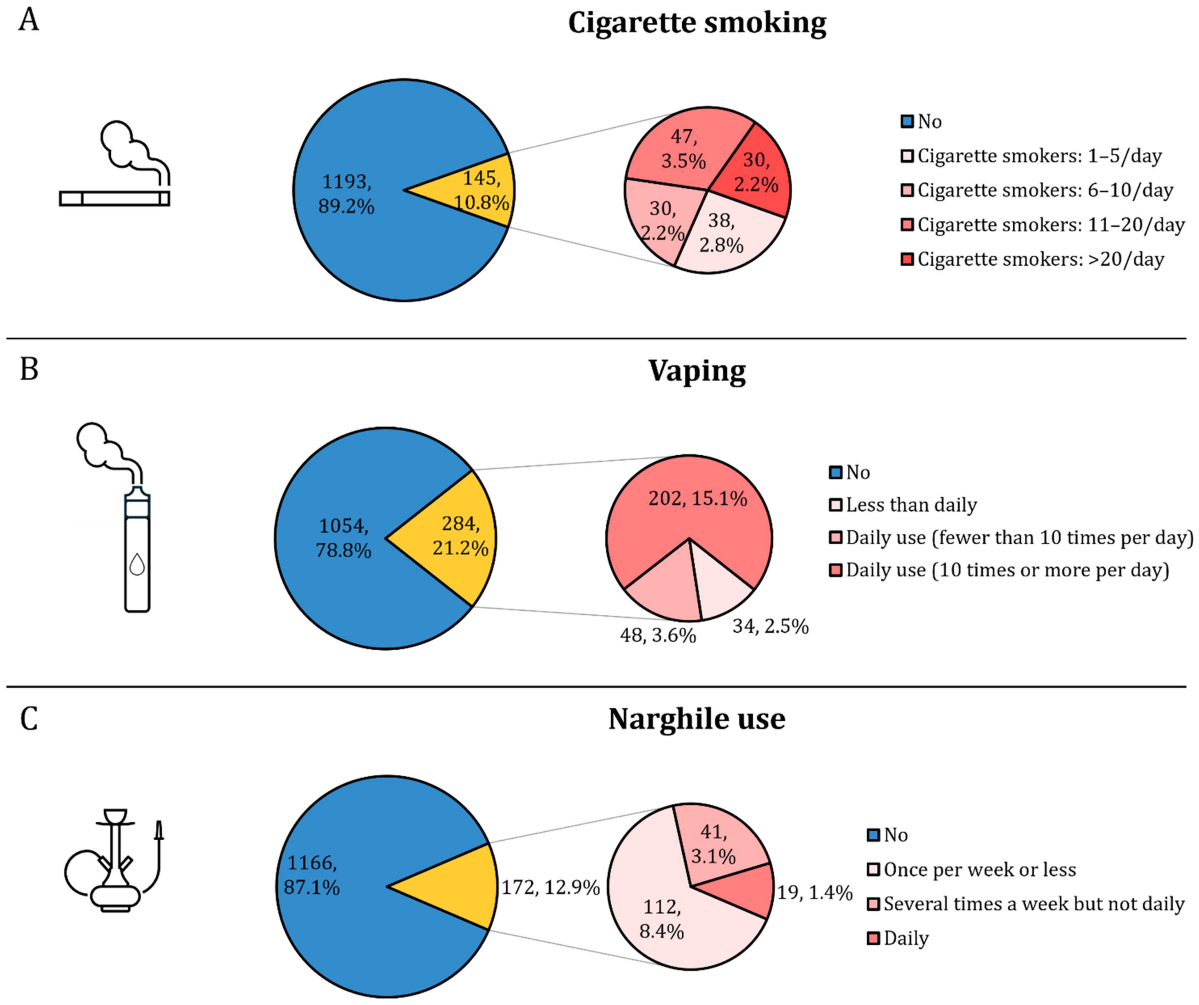
Prevalence and frequency of tobacco use among participating university students (*N* = 1,338). **(A)** Shows the prevalence and daily frequency of cigarette smoking; **(B)** shows vaping prevalence and frequency; and **(C)** shows narghile (waterpipe) use prevalence and frequency.

Among current cigarette smokers (*n* = 145), 32.4% reported smoking 11–20 cigarettes per day, while 20.7% smoked more than 20 cigarettes daily. Lower frequency use was reported by 26.2% (1–5 cigarettes) and 20.7% (6–10 cigarettes). Among vapers (*n* = 284), 71.1% reported high-frequency use (≥10 times daily), while 16.9% vaped fewer than 10 times per day, and 12.0% used less than daily. Narghile users (*n* = 172) predominantly reported infrequent use, with 65.1% smoking once per week or less, 23.8% several times per week, and 11.0% daily (Figure 2).

Significant associations were observed between demographic variables and tobacco use behaviors among the participating university students in univariate analysis. Cigarette smoking, vaping, and narghile use were all significantly more prevalent among older age groups (*p* < 0.001). Males were significantly more likely to engage in all forms of tobacco use compared to females (cigarette smoking: 22.3% vs. 2.5%; vaping: 36.9% vs. 9.7%; narghile use: 21.2% vs. 6.7%; all *p* < 0.001).

Nationality emerged as a significant factor associated with tobacco use patterns (*p* < 0.001 for all comparisons in univariate analysis). Students from the UAE nationality group reported the highest prevalence of vaping (39.6%), followed by Kuwait (24.2%) and Jordan (20.5%). In contrast, Egypt and KSA reported the lowest vaping rates (7.3 and 8.8%, respectively). Similarly, narghile use was most prevalent among students from the UAE (25.4%) and Jordan (20.1%), whereas Kuwait and KSA reported the lowest rates (3.2 and 3.8%, respectively). Cigarette smoking followed a different trend, with the highest rates observed among students from Kuwait (16.7%) and Jordan (11.2%), and the lowest in Egypt (6.0%).

Private university students reported significantly higher rates of vaping (32.4% vs. 17.6%) and narghile use (22.6% vs. 9.7%) than public university students (both *p* < 0.001). Similarly, students from scientific and humanities faculties showed higher rates of cigarette smoking compared to health students (17.2 and 21.9% vs. 7.5%, *p* < 0.001), though differences in vaping and narghile use by faculty were not statistically significant. Monthly allowance and income were associated with vaping but not consistently with cigarette or narghile use. Vaping was most common among those reporting a high monthly allowance (27.7%, *p* = 0.034) and high family income (28.1%, *p* = 0.012, Table 2).

**Table 2:** Univariate associations between demographic characteristics and current use of cigarettes, vaping, and narghile among participating university students in Arab countries.

Variable	Category	Cigarette smoking		*p* value, χ^2^	Vaping		*p* value, χ^2^	Narghile use		*p* value, χ^2^
		Yes	No		Yes	No		Yes	No	
		Count (%)	Count (%)		Count (%)	Count (%)		Count (%)	Count (%)	
Age category	18–20 years	30 (6.3)	445 (93.7)	<0.001, 47.272	70 (14.7)	405 (85.3)	<0.001, 18.875	36 (7.6)	439 (92.4)	<0.001, 19.646
	21–24 years	67 (10.1)	598 (89.9)		162 (24.4)	503 (75.6)		100 (15.0)	565 (85.0)	
	>24 years	48 (24.2)	150 (75.8)		52 (26.3)	146 (73.7)		36 (18.2)	162 (81.8)	
Sex	Male	126 (22.3)	440 (77.7)	<0.001, 132.503	209 (36.9)	357 (63.1)	<0.001, 144.615	120 (21.2)	446 (78.8)	<0.001, 61.001
	Female	19 (2.5)	753 (97.5)		75 (9.7)	697 (90.3)		52 (6.7)	720 (93.3)	
Nationality	Egypt	15 (6.0)	233 (94.0)	0.004, 17.251	18 (7.3)	230 (92.7)	<0.001, 109.485	14 (5.6)	234 (94.4)	<0.001, 128.943
	Jordan	25 (11.2)	199 (88.8)		46 (20.5)	178 (79.5)		45 (20.1)	179 (79.9)	
	Kuwait	47 (16.7)	234 (83.3)		68 (24.2)	213 (75.8)		9 (3.2)	272 (96.8)	
	KSA^c^	22 (9.2)	216 (90.8)		21 (8.8)	217 (91.2)		9 (3.8)	229 (96.2)	
	UAE^d^	16 (11.9)	118 (88.1)		53 (39.6)	81 (60.4)		34 (25.4)	100 (74.6)	
	Other^e^	20 (9.4)	193 (90.6)		78 (36.6)	135 (63.4)		61 (28.6)	152 (71.4)	
University location	Egypt	14 (5.7)	232 (94.3)	<0.001, 26.369	13 (5.3)	233 (94.7)	<0.001, 172.546	10 (4.1)	236 (95.9)	<0.001, 146.876
	Jordan	40 (11.1)	321 (88.9)		60 (16.6)	301 (83.4)		38 (10.5)	323 (89.5)	
	Kuwait	27 (23.1)	90 (76.9)		36 (30.8)	81 (69.2)		5 (4.3)	112 (95.7)	
	KSA	22 (9.0)	223 (91.0)		20 (8.2)	225 (91.8)		9 (3.7)	236 (96.3)	
	UAE	25 (10.5)	212 (89.5)		107 (45.1)	130 (54.9)		79 (33.3)	158 (66.7)	
	Other^f^	17 (12.9)	115 (87.1)		48 (36.4)	84 (63.6)		31 (23.5)	101 (76.5)	
University type	Public	103 (10.2)	908 (89.8)	0.179, 1.804	178 (17.6)	833 (82.4)	<0.001, 32.410	98 (9.7)	913 (90.3)	<0.001, 36.912
	Private	42 (12.8)	285 (87.2)		106 (32.4)	221 (67.6)		74 (22.6)	253 (77.4)	
Faculty	Health	71 (7.5)	879 (92.5)	<0.001, 40.544	186 (19.6)	764 (80.4)	0.070, 5.315	121 (12.7)	829 (87.3)	0.895, 0.222
	Scientific	40 (17.2)	193 (82.8)		59 (25.3)	174 (74.7)		32 (13.7)	201 (86.3)	
	Humanities	34 (21.9)	121 (78.1)		39 (25.2)	116 (74.8)		19 (12.3)	136 (87.7)	
Monthly family income^a^	Low	13 (14.6)	76 (85.4)	0.156, 3.722	19 (21.3)	70 (78.7)	0.012, 8.845	14 (15.7)	75 (84.3)	0.700, 0.715
	Moderate	99 (9.9)	901 (90.1)		195 (19.5)	805 (80.5)		127 (12.7)	873 (87.3)	
	High	33 (13.3)	216 (86.7)		70 (28.1)	179 (71.9)		31 (12.4)	218 (87.6)	
Monthly allowance^b^	Low	15 (9.1)	150 (90.9)	0.039, 6.506	34 (20.6)	131 (79.4)	0.034, 6.753	21 (12.7)	144 (87.3)	0.819, 0.399
	Moderate	95 (10.0)	854 (90.0)		188 (19.8)	761 (80.2)		125 (13.2)	824 (86.8)	
	High	35 (15.6)	189 (84.4)		62 (27.7)	162 (72.3)		26 (11.6)	198 (88.4)	

a Monthly family income: Self-reported by the participant.

b Monthly allowance: Self-reported by the participant.

c KSA: Kingdom of Saudi Arabia.

d UAE: United Arab Emirates.

e Other: Includes participants from Syria, Iraq, Oman, Palestine, Bahrain, Lebanon, Morocco, Qatar, Yemen, Sudan, Algeria, Tunisia, Libya, and other unspecified countries.

f Other: Includes participants studying at universities located in Bahrain, Oman, Qatar, Morocco, Lebanon, Syria, Yemen, Tunisia, Algeria, Iraq, Palestine, Sudan, Libya, and other unspecified countries.

Multinomial logistic regression was employed to identify demographic factors associated with tobacco use behaviors, including cigarette smoking, vaping, and narghile use. For cigarette smoking, the model explained 28.8% of the variance. Male students had markedly higher odds of cigarette smoking compared to females (AOR = 12.80, 95% CI: 7.52–21.80, *p* < 0.001), while younger age groups were significantly less likely to smoke than those aged >24 years (18–20 years: AOR = 0.36, 95% CI: 0.20–0.64, *p* = 0.001; 21–24 years: AOR = 0.50, 95% CI: 0.31–0.81, *p* = 0.005). Enrollment in health faculties was associated with reduced odds of smoking compared to humanities (AOR = 0.50, 95% CI: 0.27–0.91, *p* = 0.024).

For vaping behavior (Nagelkerke R^2^ = 0.349), male sex remained a significant associated factor (AOR = 6.97, 95% CI: 4.95–9.82, *p* < 0.001). Students studying in Egypt (AOR = 0.13, 95% CI: 0.04–0.41, *p* = 0.001), Jordan (AOR = 0.34, 95% CI: 0.17–0.67, *p* = 0.002), and KSA (AOR = 0.18, 95% CI: 0.03–0.96, *p* = 0.045) had lower odds of vaping compared to those in the “Other” countries category, while students in the UAE exhibited significantly higher odds (AOR = 2.31, 95% CI: 1.19–4.50, *p* = 0.013).

The narghile use multivariate model accounted for 31.4% of the variance. Male students had increased odds of narghile use (AOR = 3.95, 95% CI: 2.69–5.80, *p* < 0.001). Jordanian nationality was associated with higher odds of use (AOR = 2.30, 95% CI: 1.01–5.24, *p* = 0.048), whereas students from Kuwait (AOR = 0.17, 95% CI: 0.07–0.44, *p* < 0.001), as well as those studying in Egypt (AOR = 0.21, 95% CI: 0.06–0.75, *p* = 0.016) and Jordan (AOR = 0.28, 95% CI: 0.12–0.69, *p* = 0.005), had significantly lower odds of narghile use relative to their respective reference groups (Table 3).

**Table 3:** Multinomial logistic regression analysis of demographic variables associated with current cigarette smoking, vaping, and narghile use among university students in Arab countries.

Variable	AOR^g^ (95% CI^h^)	*p* value
Cigarette smoking (yes vs. no); Nagelkerke R^2^ = 0.288		
Age category		
18–20 years	0.362 (0.204–0.644)	**0.001**
21–24 years	0.497 (0.305–0.811)	**0.005**
>24 years	Ref.	
Sex		
Male	12.801 (7.518–21.795)	**<0.001**
Female	Ref.	
Nationality		
Egypt	1.669 (0.379–7.347)	0.498
Jordan	1.164 (0.471–2.879)	0.742
Kuwait	1.188 (0.522–2.702)	0.681
KSA^a^	3.331 (0.504–22.027)	0.212
UAE^b^	1.441 (0.552–3.763)	0.455
Other^c^	Ref.	
University location		
Egypt	0.516 (0.105–2.541)	0.416
Jordan	1.628 (0.678–3.910)	0.275
Kuwait	2.227 (0.884–5.605)	0.089
KSA	0.733 (0.113–4.774)	0.746
UAE	1.059 (0.413–2.713)	0.905
Other^d^	Ref.	
Faculty		
Health	0.500 (0.274–0.912)	**0.024**
Scientific	0.855 (0.478–1.532)	0.600
Humanities	Ref.	
Monthly allowance^e^		
Low	0.508 (0.246–1.051)	0.068
Moderate	0.696 (0.430–1.128)	0.141
High	Ref.	
Vaping (yes vs. no); Nagelkerke R^2^ = 0.349		
Age category		
18–20 years	0.963 (0.587–1.577)	0.880
21–24 years	1.307 (0.839–2.035)	0.237
>24 years	Ref.	
Sex		
Male	6.972 (4.951–9.817)	**<0.001**
Female	Ref.	
Nationality		
Egypt	0.552 (0.192–1.591)	0.271
Jordan	1.135 (0.572–2.253)	0.717
Kuwait	0.747 (0.402–1.389)	0.357
KSA	1.890 (0.347–10.293)	0.462
UAE	0.731 (0.366–1.459)	0.374
Other	Ref.	
University location		
Egypt	0.127 (0.039–0.414)	**0.001**
Jordan	0.339 (0.173–0.665)	**0.002**
Kuwait	1.208 (0.576–2.535)	0.617
KSA	0.179 (0.034–0.960)	**0.045**
UAE	2.314 (1.192–4.495)	**0.013**
Other	Ref.	
University type		
Public	0.857 (0.568–1.291)	0.460
Private	Ref.	
Faculty		
Health	1.529 (0.903–2.588)	0.114
Scientific	1.070 (0.622–1.843)	0.806
Humanities	Ref.	
Monthly family income^f^		
Low	0.667 (0.300–1.484)	0.321
Moderate	0.712 (0.463–1.097)	0.124
High	Ref.	
Monthly allowance		
Low	0.738 (0.382–1.424)	0.365
Moderate	0.656 (0.421–1.023)	0.063
High	Ref.	
Narghile use (yes vs. no); Nagelkerke R^2^ = 0.314		
Age category		
18–20 years	0.691 (0.393–1.218)	0.201
21–24 years	1.224 (0.752–1.992)	0.416
>24 years	Ref.	
Sex		
Male	3.950 (2.689–5.804)	**<0.001**
Female	Ref.	
Nationality		
Egypt	0.567 (0.185–1.732)	0.319
Jordan	2.297 (1.007–5.244)	**0.048**
Kuwait	0.174 (0.069–0.435)	**<0.001**
KSA	0.834 (0.150–4.649)	0.836
UAE	0.623 (0.312–1.244)	0.180
Other	Ref.	
University location		
Egypt	0.205 (0.056–0.747)	**0.016**
Jordan	0.283 (0.117–0.685)	**0.005**
Kuwait	0.473 (0.145–1.536)	0.213
KSA	0.219 (0.039–1.217)	0.083
UAE	1.883 (0.951–3.727)	0.069
Other	Ref.	
University type		
Public	0.672 (0.421–1.073)	0.096
Private	Ref.	

a KSA: Kingdom of Saudi Arabia.

b UAE: United Arab Emirates.

c Other: Includes participants from Syria, Iraq, Oman, Palestine, Bahrain, Lebanon, Morocco, Qatar, Yemen, Sudan, Algeria, Tunisia, Libya, and other unspecified countries.

d Other: Includes participants studying at universities located in Bahrain, Oman, Qatar, Morocco, Lebanon, Syria, Yemen, Tunisia, Algeria, Iraq, Palestine, Sudan, Libya, and other unspecified countries.

e Monthly allowance: Self-reported by the participant.

f Monthly family income: Self-reported by the participant.

g AOR: Adjusted odds ratio.

h CI: Confidence interval.

Statistically significant *p* values are highlighted in bold style.

### 3.3 Endorsement of Tobacco use attitude among the participating smokers

Among current smokers (*n* = 373), attitudes toward tobacco use were measured on a 5-point Likert scale, with higher scores indicating stronger agreement with pro-smoking beliefs. In the overall sample of smokers, the attitude was neutral leaning toward being favorable as indicated by the average ETUAS score of 3.25 ± 0.92. Male students reported significantly higher attitude scores than females (3.33 ± 0.92 vs. 3.01 ± 0.88, *p* = 0.001). Significant differences were also observed by nationality and university location, with the highest scores reported among students from the UAE and other nationalities (3.56 ± 0.81 and 3.49 ± 0.74, respectively, *p* < 0.001), and the lowest among those from Egypt (2.68 ± 0.91). University location followed a similar pattern, with Egyptian university affiliation reporting the lowest scores (2.45 ± 0.94, *p* < 0.001). No significant differences were observed across age, university type, faculty, or income levels, though students with higher allowances tended to report more favorable attitudes (*p* = 0.063) (see Table 4).

**Table 4:** Endorsement of tobacco use among the currently smoking student participants (*n* = 373) stratified per demographic variables.

Variable	Category	Average ETUAS^g^	*p* value^i^
		Mean±SD^h^	
Age category	18–20 years	3.13 ± 0.95	0.397
	21–24 years	3.32 ± 0.87	
	>24 years	3.23 ± 1.01	
Sex	Male	3.33 ± 0.92	0.001
	Female	3.01 ± 0.88	
Nationality	Egypt	2.68 ± 0.91	<0.001
	Jordan	2.97 ± 0.95	
	Kuwait	3.23 ± 0.92	
	KSA^c^	3.27 ± 1.13	
	UAE^d^	3.56 ± 0.81	
	Other^e^	3.49 ± 0.74	
University location	Egypt	2.45 ± 0.94	<0.001
	Jordan	2.99 ± 0.93	
	Kuwait	3.29 ± 0.87	
	KSA	3.32 ± 1.15	
	UAE	3.44 ± 0.82	
	Other^f^	3.58 ± 0.67	
University type	Public	3.31 ± 0.93	0.117
	Private	3.15 ± 0.91	
Faculty	Health	3.20 ± 0.90	0.519
	Scientific	3.34 ± 0.96	
	Humanities	3.33 ± 0.97	
Monthly family income^a^	Low	3.01 ± 1.04	0.449
	Moderate	3.25 ± 0.91	
	High	3.32 ± 0.91	
Monthly allowance^b^	Low	3.42 ± 0.98	0.063
	Moderate	3.18 ± 0.91	
	High	3.38 ± 0.93	

a Monthly family income: Self-reported by the participant.

b Monthly allowance: Self-reported by the participant.

c KSA: Kingdom of Saudi Arabia.

d UAE: United Arab Emirates.

e Other: Includes participants from Syria, Iraq, Oman, Palestine, Bahrain, Lebanon, Morocco, Qatar, Yemen, Sudan, Algeria, Tunisia, Libya, and other unspecified countries.

f Other: Includes participants studying at universities located in Bahrain, Oman, Qatar, Morocco, Lebanon, Syria, Yemen, Tunisia, Algeria, Iraq, Palestine, Sudan, Libya, and other unspecified countries.

g ETUAS: Endorsement of Tobacco Use Attitude Score.

h SD: Standard deviation.

i *p* value: calculated using Mann–Whitney *U* (M-W) test and Kruskal–Wallis H (K-W) test.

In the multinomial logistic regression model for the factors associated with endorsement of tobacco use attitude (ETUAS, Nagelkerke R^2^ = 0.254), being male was significantly associated with a favorable attitude toward tobacco use compared to an unfavorable one (AOR: 4.29, 95% CI: 1.89–9.71, *p* < 0.001). Students studying in Egypt (AOR: 0.013, 95% CI: 0.001–0.237, *p* = 0.003), Jordan (AOR: 0.034, 95% CI: 0.004–0.331, *p* = 0.004), or Kuwait (AOR: 0.070, 95% CI: 0.007–0.731, *p* = 0.026) had significantly lower odds of neutral attitudes compared to unfavorable, relative to students in “Other” Arab countries category. Additionally, a low monthly allowance was significantly associated with lower odds of neutral versus unfavorable attitudes (AOR: 0.245, 95% CI: 0.068–0.891, *p* = 0.033, Table 5).

**Table 5:** Multinomial logistic regression analysis of factors associated with attitude toward tobacco use (endorsement of tobacco use attitude score (ETUAS) categories: neutral and favorable vs. unfavorable) among university students.

ETUAS category, Nagelkerke R^2^ = 0.254	Neutral vs. unfavorable		Favorable vs. unfavorable	
	AOR^f^ (95% CI^g^)	*p* value	AOR 7 (95% CI 8)	*p* value
Sex				
Male	1.280 (0.632–2.594)	0.493	4.286 (1.892–9.709)	**<0.001**
Female	Ref.		Ref.	
Nationality				
Egypt	1.191 (0.203–6.993)	0.846	0.345 (0.045–2.647)	0.306
Jordan	1.234 (0.344–4.425)	0.747	0.443 (0.112–1.757)	0.247
Kuwait	0.838 (0.218–3.223)	0.797	0.549 (0.135–2.226)	0.401
KSA^a^	2.284 (0.091–57.291)	0.615	0.356 (0.012–10.242)	0.547
UAE^b^	1.406 (0.347–5.700)	0.634	1.854 (0.450–7.647)	0.393
Other^c^	Ref.		Ref.	
University location				
Egypt	0.018 (0.001–0.267)	**0.004**	0.013 (0.001–0.237)	**0.003**
Jordan	0.042 (0.004–0.391)	**0.005**	0.034 (0.004–0.331)	**0.004**
Kuwait	0.070 (0.007–0.731)	**0.026**	0.104 (0.010–1.094)	0.059
KSA	0.032 (0.001–1.373)	0.073	0.274 (0.006–12.584)	0.507
UAE	0.121 (0.014–1.083)	0.059	0.123 (0.014–1.124)	0.063
Other^d^	Ref.		Ref.	
Monthly allowance^e^				
Low	0.245 (0.068–0.891)	**0.033**	0.587 (0.162–2.129)	0.418
Moderate	0.500 (0.222–1.125)	0.094	0.481 (0.202–1.147)	0.099
High	Ref.		Ref.	

a KSA: Kingdom of Saudi Arabia.

b UAE: United Arab Emirates.

c Other: includes participants from Syria, Iraq, Oman, Palestine, Bahrain, Lebanon, Morocco, Qatar, Yemen, Sudan, Algeria, Tunisia, Libya, and other unspecified countries.

d Other: includes participants studying at universities located in Bahrain, Oman, Qatar, Morocco, Lebanon, Syria, Yemen, Tunisia, Algeria, Iraq, Palestine, Sudan, Libya, and other unspecified countries.

e Monthly allowance: self-reported by the participant.

f AOR: Adjusted odds ratio.

g CI: Confidence interval.

Statistically significant *p* values are highlighted in bold style.

### 3.4 Psychological determinants of vaping

Table 6 presents the mean scores for the four m-VAPeS constructs across key demographic and ETUAS subgroups. Social Influence scores differed significantly by nationality (*p* < 0.001), university location (*p* < 0.001), and monthly allowance (*p* = 0.010). Students from the UAE and those with lower allowances reported the highest scores. ETUAS categories also showed significant differences (*p* < 0.001), with favorable attitudinal groups scoring highest. Perceived Benefits varied by nationality (*p* = 0.008) and university location (*p* = 0.008), with UAE-based and “Other nationality” students reporting higher scores. A significant difference was also observed by ETUAS category (*p* = 0.028). Behavioral Influence - Risk was significantly associated with sex (*p* = 0.026), nationality (*p* < 0.001), university location (*p* < 0.001), and monthly allowance (*p* = 0.013). Females and participants with low allowances had higher scores, and students from KSA reported the highest levels. Behavioral Influence - Situational Triggers scores varied significantly by age (*p* = 0.046), sex (*p* = 0.024), nationality (*p* = 0.045), university location (*p* = 0.002), and ETUAS category (*p* = 0.006). Older students, males, and those with favorable tobacco attitudes exhibited higher scores.

**Table 6:** Mean scores for the four modified VAPeS constructs—Social Influence (SI), Perceived Benefits (BP), Behavioral Influence - Risk (BI-R), and Behavioral Influence—Situational Triggers (BI-ST)—stratified by demographic characteristics and Endorsement of Tobacco Use Attitude Score (ETUAS) categories among students who indicated tobacco use (*n* = 373).

Variable	Category	Average SI	*p* value^g^	Average BP	*p* value	Average BI-R	*p* value	Average BI-ST	*p* value
		Mean ± SD^h^		Mean ± SD		Mean ± SD		Mean ± SD	
Age category	18–20 years	2.70 ± 1.04	0.061	3.41 ± 0.92	0.949	1.92 ± 0.91	0.587	4.09 ± 0.86	0.046
	21–24 years	2.72 ± 0.89		3.44 ± 0.89		1.92 ± 0.79		4.19 ± 0.78	
	>24 years	2.43 ± 0.87		3.35 ± 1.14		2.00 ± 0.74		3.96 ± 0.69	
Sex	Male	2.66 ± 0.95	0.656	3.48 ± 0.94	0.066	1.87 ± 0.78	0.026	4.19 ± 0.74	0.024
	Female	2.68 ± 0.88		3.23 ± 0.91		2.13 ± 0.85		3.92 ± 0.89	
Nationality	Egypt	2.43 ± 0.86	<0.001	3.35 ± 0.87	0.008	2.19 ± 0.89	<0.001	3.93 ± 0.63	0.045
	Jordan	2.62 ± 0.95		3.36 ± 0.98		1.75 ± 0.74		4.17 ± 0.74	
	Kuwait	2.07 ± 0.93		3.11 ± 1.00		1.60 ± 0.88		3.90 ± 1.00	
	KSA^c^	2.43 ± 0.95		3.05 ± 1.24		2.36 ± 1.00		3.78 ± 1.09	
	UAE^d^	3.09 ± 0.64		3.68 ± 0.77		2.10 ± 0.66		4.32 ± 0.54	
	Other^e^	3.03 ± 0.79		3.65 ± 0.80		2.05 ± 0.68		4.29 ± 0.59	
University location	Egypt	1.92 ± 0.75	<0.001	3.44 ± 0.84	0.008	1.85 ± 0.80	<0.001	3.79 ± 0.66	0.002
	Jordan	2.28 ± 0.96		3.19 ± 0.99		1.58 ± 0.71		4.05 ± 0.79	
	Kuwait	2.30 ± 1.05		3.07 ± 1.04		1.78 ± 1.00		3.72 ± 1.10	
	KSA	2.44 ± 1.00		3.15 ± 1.17		2.42 ± 0.98		3.77 ± 1.08	
	UAE	3.09 ± 0.67		3.68 ± 0.81		2.07 ± 0.70		4.35 ± 0.57	
	Other^f^	2.76 ± 0.87		3.47 ± 0.86		2.03 ± 0.76		4.24 ± 0.63	
University type	Public	2.65 ± 0.96	0.904	3.42 ± 0.91	0.895	1.98 ± 0.81	0.206	4.12 ± 0.74	0.655
	Private	2.69 ± 0.88		3.41 ± 0.99		1.86 ± 0.81		4.12 ± 0.87	
Faculty	Health	2.68 ± 0.87	0.407	3.47 ± 0.88	0.123	1.95 ± 0.79	0.442	4.19 ± 0.71	0.392
	Scientific	2.74 ± 1.00		3.41 ± 0.99		1.87 ± 0.92		3.99 ± 0.87	
	Humanities	2.49 ± 1.07		3.15 ± 1.12		1.96 ± 0.73		3.99 ± 0.96	
Monthly family income^a^	Low	2.78 ± 0.84	0.435	3.49 ± 0.81	0.642	2.11 ± 0.84	0.107	4.21 ± 0.79	0.240
	Moderate	2.68 ± 0.91		3.38 ± 0.93		1.97 ± 0.81		4.07 ± 0.79	
	High	2.59 ± 1.01		3.5 ± 1.02		1.79 ± 0.77		4.23 ± 0.78	
Monthly allowance^b^	Low	2.83 ± 0.86	0.010	3.59 ± 0.71	0.419	2.15 ± 0.73	0.013	4.24 ± 0.76	0.591
	Moderate	2.72 ± 0.91		3.43 ± 0.95		1.97 ± 0.82		4.11 ± 0.76	
	High	2.40 ± 0.99		3.29 ± 1.01		1.73 ± 0.79		4.09 ± 0.88	
ETUAS category	Unfavorable	2.02 ± 0.76	<0.001	3.30 ± 1.06	0.028	1.81 ± 1.06	0.104	3.80 ± 1.00	0.006
	Neutral	2.61 ± 0.85		3.30 ± 0.89		2.01 ± 0.76		4.06 ± 0.78	
	Favorable	2.93 ± 0.95		3.59 ± 0.94		1.90 ± 0.77		4.29 ± 0.68	

a Monthly family income: self-reported by the participant.

b Monthly allowance: self-reported by the participant.

c KSA: Kingdom of Saudi Arabia.

d UAE: United Arab Emirates.

e Other: includes participants from Syria, Iraq, Oman, Palestine, Bahrain, Lebanon, Morocco, Qatar, Yemen, Sudan, Algeria, Tunisia, Libya, and other unspecified countries.

f Other: includes participants studying at universities located in Bahrain, Oman, Qatar, Morocco, Lebanon, Syria, Yemen, Tunisia, Algeria, Iraq, Palestine, Sudan, Libya, and other unspecified countries.

g SD: standard deviation.

h *p* value: calculated using Mann–Whitney *U* (M-W) test and Kruskal–Wallis H (K-W) test.

### 3.5 Attitude to tobacco use among vaping users

Among current vaping users (*n* = 284), ETUAS scores varied significantly by sex, nationality, and university location. Male students were more likely to express favorable attitudes compared to females (42.1% vs. 21.7%, *p* = 0.002). Favorable attitudes were most prevalent among students from the UAE (50.9%) and other nationalities (48.3%), whereas students from Egypt demonstrated the highest percentage of unfavorable attitudes (41.4%, *p* < 0.001). Similarly, university location was significantly associated with ETUAS: students studying in Egypt exhibited the highest percentage of unfavorable attitudes (50.0%), while those studying in the UAE and “Other” locations showed higher rates of favorable attitudes (43.0 and 52.6%, respectively, *p* < 0.001). No statistically significant differences were observed by age, university type, faculty, family income, or monthly allowance (Table 7).

**Table 7:** Association of endorsement of tobacco use score (ETUAS) categories with sociodemographic characteristics among current vaping users.

Variable	Category	ETUAS Category			*p* value, χ^2^
		Unfavorable	Neutral	Favorable	
		Count (%)	Count (%)	Count (%)	
Age category	18–20 years	18 (22.5)	35 (43.8)	27 (33.8)	0.569, 2.936
	21–24 years	31 (15.6)	92 (46.2)	76 (38.2)	
	>24 years	20 (22.0)	37 (40.7)	34 (37.4)	
Sex	Male	49 (17.6)	112 (40.3)	117 (42.1)	0.002, 12.466
	Female	20 (21.7)	52 (56.5)	20 (21.7)	
Nationality	Egypt	12 (41.4)	13 (44.8)	4 (13.8)	<0.001, 38.310
	Jordan	21 (28.8)	38 (52.1)	14 (19.2)	
	Kuwait	18 (20.2)	37 (41.6)	34 (38.2)	
	KSA^c^	6 (17.1)	15 (42.9)	14 (40.0)	
	UAE^d^	4 (7.3)	23 (41.8)	28 (50.9)	
	Other^e^	8 (9.0)	38 (42.7)	43 (48.3)	
University location	Egypt	12 (50.0)	9 (37.5)	3 (12.5)	<0.001, 51.404
	Jordan	30 (31.6)	45 (47.4)	20 (21.1)	
	Kuwait	8 (17.4)	18 (39.1)	20 (43.5)	
	KSA	6 (17.6)	13 (38.2)	15 (44.1)	
	UAE	12 (10.5)	53 (46.5)	49 (43.0)	
	Other^f^	1 (1.8)	26 (45.6)	30 (52.6)	
University type	Public	41 (17.6)	99 (42.5)	93 (39.9)	0.322, 2.268
	Private	28 (20.4)	65 (47.4)	44 (32.1)	
Faculty	Health	46 (19.7)	107 (45.9)	80 (34.3)	0.675, 2.330
	Scientific	13 (16.7)	34 (43.6)	31 (39.7)	
	Humanities	10 (16.9)	23 (39.0)	26 (44.1)	
Monthly family income^a^	Low	8 (28.6)	10 (35.7)	10 (35.7)	0.548, 3.061
	Moderate	49 (19.0)	115 (44.6)	94 (36.4)	
	High	12 (14.3)	39 (46.4)	33 (39.3)	
Monthly allowance^b^	Low	7 (17.5)	11 (27.5)	22 (55.0)	0.069, 8.710
	Moderate	51 (20.4)	115 (46.0)	84 (33.6)	
	High	11 (13.8)	38 (47.5)	31 (38.8)	

a Monthly family income: self-reported by the participant.

b Monthly allowance: self-reported by the participant.

c KSA: Kingdom of Saudi Arabia.

d UAE: United Arab Emirates.

e Other: includes participants from Syria, Iraq, Oman, Palestine, Bahrain, Lebanon, Morocco, Qatar, Yemen, Sudan, Algeria, Tunisia, Libya, and other unspecified countries.

f Other: Includes participants studying at universities located in Bahrain, Oman, Qatar, Morocco, Lebanon, Syria, Yemen, Tunisia, Algeria, Iraq, Palestine, Sudan, Libya, and other unspecified countries.

In the linear regression model for the associated factors with endorsement of tobacco use among current vaping users, the overall model was statistically significant (*F*(7, 276) = 10.87; *p* < 0.001), accounting for 21.6% of the variance in attitude scores (R^2^ = 0.216; adjusted R^2^ = 0.196). Male sex was significantly associated with a more favorable attitude (*B* = −0.325; 95% CI: −0.538 to −0.112; *p* = 0.003). Higher Social Influence (*B* = 0.300; 95% CI: 0.191 to 0.410; *p* < 0.001) and greater Behavioral Influence - Situational Triggers (*B* = 0.205; 95% CI: 0.078 to 0.332; *p* = 0.002) were also significant positive associated factors. Monthly financial allowance also emerged as an associated factor (*B* = 0.199; 95% CI: 0.036 to 0.363; *p* = 0.017), indicating a potential socio-economic component in shaping attitude to tobacco use (Table 8).

**Table 8:** Multivariable linear regression analysis examining associated factors with endorsement of tobacco use among current vaping users (*n* = 284).

Dependent variable: ETUAS^a^ category Model R^2^ = 0.216	Unstandardized coefficients		Standardized coefficients	*p* value	VIF^c^
	*B*	95.0% CI^b^ for *B*	*β*		
Sex	–0.325	−0.538 to −0.112	−0.165	0.003	1.071
Nationality	0.055	−0.019 to 0.129	0.106	0.143	1.819
University location	0.027	−0.053 to 0.107	0.048	0.510	1.907
Monthly allowance	0.199	0.036 to 0.363	0.132	0.017	1.067
Average social influence score	0.300	0.191 to 0.410	0.321	<0.001	1.251
Average perceived benefits score	−0.089	−0.198 to 0.019	−0.097	0.106	1.257
Average behavioral influence - situational trigger score	0.205	0.078 to 0.332	0.186	0.002	1.208

a ETUAS: Endorsement of Tobacco Use Attitude Score.

b CI: Confidence interval.

c VIF: Variance inflation factor.

## 4 Discussion

This study represents one of the more geographically inclusive efforts to examine tobacco use attitudes among university students in the Arab region. By adopting a multi-country approach—including participants from Egypt, Jordan, Kuwait, KSA, the UAE, among Arab countries—we aimed to reflect the MENA region’s sociocultural and institutional diversity. This broader scope contributes to the contextual relevance and potential generalizability of the findings, particularly in light of the varying regulatory and cultural backgrounds that influence tobacco-related behaviors (87, 88). In this study, methodological rigor was upheld through QC measures such as limiting responses to one per IP address and incorporating internal consistency checks to identify and exclude contradictory responses which added further rigor by avoiding a common caveat of survey studies as noted by (89). While no single study can capture all dimensions of a complex public health issue, our approach was designed to balance scalability with data reliability to address the limitations in tobacco survey-based research in the MENA previously outlined by (90). In doing so, we aimed to contribute reliable evidence to the scientific discourse on youth tobacco use and to support contextually informed public health strategies in the MENA region and beyond.

In the current study, the observed prevalence of vaping among university students was 21.2%. This result is notably higher than the global average of 10.2% for current vaping use among school and college students, as reported in a recent meta-analysis encompassing 146 studies across 53 countries (91). Regionally, our estimate of vaping prevalence surpasses the 18.1% prevalence reported among 1,002 Palestinian university students (92), and aligns with findings from the UAE, where a study identified a 23% prevalence of e-cigarette use among university students during the year 2021 (28). The high prevalence of vaping in our sample, particularly among students from the UAE and Kuwait, underlines a concerning trend in the Arab region (93).

The rise of vaping among university students in this study—overtaking both cigarette and narghile use—might reflect a confluence of sociocultural, economic, and psychological determinants that merit deep consideration. The rise of vaping in youth populations might be underpinned by a fundamental shift in risk perception (94, 95). Multiple studies have documented that vaping is widely viewed as a safer alternative to combustible tobacco products in various geographic and cultural settings (95). For example, (96) found that among youth aged 13–20 years across 30 U.S. cities, past-month nicotine vaping was associated with lower perceived risks of e-cigarette use. Similarly, data from the 2016–2017 Canadian Student Tobacco, Alcohol, and Drugs Survey showed that students perceiving high risk from both cigarettes and e-cigarettes were more likely to vape than to smoke only cigarettes (97). In Thailand, a university-based study also revealed that students with lower risk perceptions were significantly more likely to use e-cigarettes (98). This low-risk perception comes despite accumulating evidence linking vaping to cardiovascular disease and long-term pulmonary injury (62, 99, 100).

The demographic variations in tobacco use behaviors observed in this study among Arab university students likely stem from an interplay of social, cultural, and regulatory factors. In this study, male students exhibited approximately thirteenfold higher odds of cigarette smoking, sevenfold higher odds of vaping, and fourfold higher odds of narghile use compared to females. This pattern aligns with longstanding gender norms in many societies where male tobacco use is socially tolerated or even valued as an attribute of masculinity (101–104). This disparity may also reflect greater exposure to peer influence, masculine identity signaling, and relative immunity from familial or institutional sanctions (105, 106). Conversely, female students may experience stronger sociocultural deterrents against visible tobacco use (107). However, the reported 9.7% prevalence of current vaping among female students who participated in this study warrants particular attention. Emerging evidence suggests that vaping products may disproportionately appeal to females due to enhanced flavor perception, higher reward sensitivity, and the discreet nature of use as delineated by several studies (108–110). These factors, coupled with targeted marketing and perceived lower harm, may be eroding historical gender gaps in tobacco use, which highlights the need for gender-responsive prevention strategies as suggested by (111).

Additionally, the inverse association between younger age and cigarette use, and the possible protective role of enrollment in health faculties, support hypotheses that growing awareness of health risks—particularly among educated youth—may be limiting traditional smoking behaviors (112). However, the same deterrents appear less effective for vaping, suggesting that e-cigarettes might be perceived as safer or more socially acceptable alternative for tobacco consumption (113, 114). This was evident in this study, especially in countries like the UAE where odds of vaping use were found to be higher in this study. This can point to an area where policy and regulatory action can help aggressive marketing may allow vaping to flourish under the guise of harm reduction (115, 116).

Among current tobacco users in this study, the attitudes toward tobacco use leaned moderately favorable (ETUAS mean = 3.25). The finding that male university students exhibited significantly more favorable attitudes toward tobacco use than their female counterparts echoes a long-standing reality in many other settings globally, where masculinity remains entangled with risk-taking, independence, and resistance to regulation—tobacco being a symbol of all three (103). In contrast, female tobacco use is often stigmatized explaining their less favorable attitude towards tobacco use in this study (107). Moreover, variability in attitude toward tobacco use based on university location could reflect differing levels of national policies targeting tobacco use control (25, 90). Furthermore, the association between low personal allowance and less neutral attitudes to tobacco use hints at an economic threshold beneath which students may see tobacco either as an unaffordable luxury or a redundant expenditure leading to more definitive rejection rather than passive ambivalence. In this light, economic constraints, paradoxically, may serve a protective function in modulating tobacco use behaviors in the absence of regulatory frameworks (117).

In this study, the use of VAPeS constructs helped to dissect the psychosocial perspectives that underpins attitude toward vaping in the Arab university context. Our findings offer a revealing window into the attitudinal architecture of vaping among Arab university students, particularly through the lens of male sex, monthly allowance, social influence, and situational triggers—each emerging as significant factors associated with favorable attitudes toward tobacco use. The strong positive association between male sex and pro-vaping attitudes may be rooted not merely in cultural permissiveness but also in broader gender norms prevalent across Arab societies, as stated earlier in the discussion. This echoes literature noting higher prevalence and endorsement of tobacco among men in the Middle East (26, 33, 118–121).

Equally salient was the role of social influence, which points to the relational nature of vaping uptake. In environments where peer dynamics and digital ecosystems amplify behavior modeling—through platforms like Instagram, TikTok, and YouTube—perceptions of social acceptance are often internalized more rapidly than clinical risk (122–124). This social contagion effect is particularly virulent among young adults navigating identity, belonging, and visibility in collectivist cultures (125, 126). Moreover, the emergence of “Behavioral Influence - Situational Triggers” as a significant factor associated with favorable attitudes toward tobacco use highlights the contextual and affective dimensions of vaping behavior. This modified VAPeS construct captured academic stress, stress relief, and ease of access to e-cigarettes and it suggests that vaping can be embedded in coping mechanisms and environmental availability. In high-stress academic settings, students may turn to vaping as a form of immediate psychological relief, with nicotine use becoming functionally intertwined with stress management routines (127–129). The accessibility of e-cigarettes further lowers the threshold for such behavior, reinforcing use through convenience and habituation (130). Additionally, the independent association between higher monthly allowance and favorable attitudes toward tobacco use may reflect greater purchasing power and autonomy, facilitating exposure to and experimentation with vaping and tobacco products (131). This economic factor could also correlate with increased exposure to lifestyle marketing, peer networks where vaping is prevalent, or environments where vaping products are more easily accessible. The harm perception inferred through the Perceived Benefits construct was not statistically associated with attitude to tobacco use which could suggest a cognitive dissonance whereby users compartmentalize risk while foregrounding hedonic reward (132). Such findings necessitate a recalibration of intervention strategies—away from purely risk-based messaging toward those that confront the affective, social, and symbolic dimensions of vaping.

Based on our findings, we recommend a multidimensional strategy tailored to the specific behavioral, demographic, and cultural factors influencing tobacco use in Arab university populations. First, the disproportionately high odds of cigarette smoking, vaping, and narghile use among male students highlight the urgent need for gender-targeted interventions as highlighted by (133, 134). Public health campaigns should challenge masculinity norms that equate tobacco use with strength, independence, or modernity, while offering alternative models of male identity rooted in health and well-being. Second, the significantly higher prevalence of vaping compared to other tobacco products—particularly among students in the UAE and those with favorable social influence and situational triggers—calls for regulation of digital marketing (135–138). Interventions should focus on countering the glamorization of vaping through peer-led education and media literacy programs (139, 140). Third, the inverse association between enrollment in health-related faculties and smoking suggests that education can be a protective factor. Thus, integrating comprehensive tobacco education into university curricula and enforcing a tobacco-free campus policies may foster more critical attitudes toward tobacco use (141). Fourth, the finding that students with lower allowances had less favorable attitudes suggests that socioeconomic vulnerability may serve as a buffer, possibly due to financial constraints or prioritization of essential expenses. Public health policies could leverage this by introducing taxation or pricing policies that render vaping and tobacco products economically inaccessible to students (142). Fifth, country-specific disparities in tobacco behavior and attitudes highlight the importance of tailoring national policies and campus-level interventions to local cultural and regulatory contexts rather than adopting a one-size-fits-all model. This regional variation in policies was recently reported by (143). Finally, future research should move beyond association-based models to explore potential mediating and moderating pathways particularly those grounded in behavioral theory frameworks such as the TPB or the Health Belief Model (HBM). Such an approach would be helpful to clarify the psychosocial mechanisms driving tobacco and vaping behaviors among youth. Additionally, future research should incorporate more granular assessments of national tobacco control policies and socio-cultural contexts to better contextualize behavioral findings and guide country-specific public health interventions.

Finally, this study, while multinational and methodologically robust, was not without limitations. First, the use of convenience and snowball sampling limits the generalizability of the findings. Participants were self-selected and may not be representative of the wider university student populations within each country, particularly given potential overrepresentation from health faculties and public universities. Notably, 71% of respondents were from health-related faculties and 76% from public universities, which may skew the findings toward the attitudes, knowledge, and behaviors prevalent in those sub-groups. In light of the sampling strategy employed, statistical inferences including CIs and *p* values should be interpreted descriptively, and not as representative of national or regional prevalence estimates. Second, the reliance on self-reported data introduces the possibility of information bias, particularly social desirability bias, which may have led to underreporting of tobacco use or overreporting of socially acceptable attitudes. Third, the cross-sectional design precludes any causal inference between sociodemographic factors, psychosocial constructs, and tobacco-related behaviors or attitudes. Fourth, although the study included participants from several Arab countries, only five nationalities reached the minimum sample size threshold for country-specific analyses, thereby limiting the granularity and cross-national comparability of results. Participants from remaining Arab countries were grouped under “Other” category to preserve regional breadth of the study, with findings reported descriptively in acknowledgment of the group’s heterogeneity. Fifth, while the VAPeS scale was validated for use among Arabic-speaking university students, the psychometric performance of its sub-scales may vary across cultural contexts, potentially affecting the interpretation of construct scores. Additionally, while most VAPeS sub-scales demonstrated high internal consistency, the Economic and Self-Efficacy sub-scale showed a comparatively lower Cronbach’s *α*, which remains acceptable for exploratory research but warrants cautious interpretation if considered in isolation. Sixth, attitudes were assessed using a novel four-item scale derived from WHO-recommended items, yet the measure of ETUAS was not benchmarked against broader theoretical models or long-term behavioral outcomes. Finally, although we employed multivariable modeling to explore associations between psychosocial factors and tobacco use behaviors, these models are exploratory in nature and not intended to support predictive generalizations beyond the study sample.

## 5 Conclusion

This multinational cross-sectional study revealed a concerning pattern of tobacco use among university students across the Arab world, with vaping emerging as the most prevalent modality, surpassing both cigarette smoking and narghile use. Male students were consistently more likely to engage in all forms of tobacco use, and among current users, males exhibited significantly more favorable attitudes toward continued use. Regional disparities were notable, with UAE students reporting markedly higher vaping rates, while students in Egypt and Jordan were more likely to hold unfavorable attitudes toward smoking. Multivariate analyses further highlighted the role of social influence, behavioral situational triggers (e.g., stress, ease of access), and higher monthly allowance as key associated factors with pro-tobacco attitudes, particularly among male vapers. The findings highlighted the need for culturally tailored public health interventions that account for sociocultural drivers such as peer influence, stress-related vaping behaviors, and access regulation. Educational campaigns must address the rising social acceptability of vaping, particularly in affluent student groups and private university contexts, where usage is disproportionately high. Regulatory frameworks should extend beyond cigarettes to include electronic nicotine delivery systems, particularly in countries with rising uptake among youth. Finally, the m-VAPeS scale proved effective in capturing behavioral and perceptual dimensions of vaping and can be further utilized for targeted research and policymaking in similar populations. As the Arab region stands at the heart of a vaping epidemic, the study findings provide a timely evidence base to inform comprehensive, evidence-driven tobacco control strategies in the region.

## Data Availability

The datasets presented in this study can be found in online repositories. The names of the repository/repositories and accession number(s) can be found in the article/Supplementary material.

## References

[1] Hanafin J Clancy L. History of Tobacco production and use In: Karger S, editor. The Tobacco epidemic (2015)

[2] Hooker C. Learning to smoke: tobacco use in the west. Tob Control. (2003) 12:340. doi: 10.1136/tc.12.3.340

[3] Samet JM. Tobacco smoking: the leading cause of preventable disease worldwide. Thorac Surg Clin. (2013) 23:103–12. doi: 10.1016/j.thorsurg.2013.01.009, PMID: 23566962

[4] Banks E Joshy G Korda RJ Stavreski B Soga K Egger S. Tobacco smoking and risk of 36 cardiovascular disease subtypes: fatal and non-fatal outcomes in a large prospective Australian study. BMC Med. (2019) 17:128. doi: 10.1186/s12916-019-1351-4, PMID: 31266500 PMC6607519

[5] Fang M Xia Z Rong X Xiao J. The association of smoking on the increased risk of osteoporotic fracture: results from a cross-sectional study and two-sample Mendelian randomization. Tob Induc Dis. (2024) 22:1–11. doi: 10.18332/tid/189485, PMID: 38933524 PMC11201227

[6] Liu Y Lu L Yang H Wu X Luo X Shen J. Dysregulation of immunity by cigarette smoking promotes inflammation and cancer: a review. Environ Pollut. (2023) 339:122730. doi: 10.1016/j.envpol.2023.122730, PMID: 37838314

[7] Lu W Aarsand R Schotte K Han J Lebedeva E Tsoy E. Tobacco and COPD: presenting the World Health Organization (WHO) Tobacco knowledge summary. Respir Res. (2024) 25:338. doi: 10.1186/s12931-024-02961-5, PMID: 39261873 PMC11391604

[8] Velilla S García-Medina JJ García-Layana A Dolz-Marco R Pons-Vázquez S Pinazo-Durán MD. Smoking and age-related macular degeneration: review and update. J Ophthalmol. (2013) 2013:895147. doi: 10.1155/2013/895147, PMID: 24368940 PMC3866712

[9] Warren GW Cummings KM. Tobacco and lung cancer: risks, trends, and outcomes in patients with cancer. Am Soc Clin Oncol Educ Book. (2013) 359-364:359–64. doi: 10.14694/EdBook_AM.2013.33.359, PMID: 23714547

[10] Zhu S Gao J Zhang L Dong W Shi W Guo H. Global, regional, and national cardiovascular disease burden attributable to smoking from 1990 to 2021: findings from the GBD 2021 study. Tob Induc Dis. (2025) 23:1–12. doi: 10.18332/tid/200072, PMID: 39897459 PMC11784507

[11] GBD 2019 Tobacco Collaborators. Spatial, temporal, and demographic patterns in prevalence of smoking tobacco use and attributable disease burden in 204 countries and territories, 1990-2019: a systematic analysis from the global burden of disease study 2019. Lancet. (2021) 397:2337–60. doi: 10.1016/s0140-6736(21)01169-7, PMID: 34051883 PMC8223261

[12] Le Foll B Piper ME Fowler CD Tonstad S Bierut L Lu L. Tobacco and nicotine use. Nat Rev Dis Prim. (2022) 8:19. doi: 10.1038/s41572-022-00346-w, PMID: 35332148

[13] O'Connor R Schneller L Felicione N Schneller LM Felicione NJ Talhout R. Evolution of tobacco products: recent history and future directions. Tob Control. (2022) 31:175–82. doi: 10.1136/tobaccocontrol-2021-056544, PMID: 35241585

[14] World Health Organization (2021). WHO report on the global tobacco epidemic, 2021: addressing new and emerging products. Available online at: https://iris.who.int/handle/10665/343287 (Accessed April 20, 2025).

[15] Li Y Hecht SS. Carcinogenic components of tobacco and tobacco smoke: a 2022 update. Food Chem Toxicol. (2022) 165:113179. doi: 10.1016/j.fct.2022.113179, PMID: 35643228 PMC9616535

[16] Talhout R Schulz T Florek E van Benthem J Wester P Opperhuizen A. Hazardous compounds in tobacco smoke. Int J Environ Res Public Health. (2011) 8:613–28. doi: 10.3390/ijerph8020613, PMID: 21556207 PMC3084482

[17] Bafunno D Catino A Lamorgese V del Bene G Longo V Montrone M. Impact of tobacco control interventions on smoking initiation, cessation, and prevalence: a systematic review. J Thorac Dis. (2020) 12:3844–56. doi: 10.21037/jtd.2020.02.23, PMID: 32802466 PMC7399441

[18] Perez-Warnisher MT De Miguel M Seijo LM. Tobacco use worldwide: legislative efforts to curb consumption. Ann Glob Health. (2018) 84:571–9. doi: 10.9204/aogh.2362, PMID: 30779502 PMC6748295

[19] Akter S Rahman MM Rouyard T Aktar S Nsashiyi RS Nakamura R. A systematic review and network meta-analysis of population-level interventions to tackle smoking behaviour. Nat Hum Behav. (2024) 8:2367–91. doi: 10.1038/s41562-024-02002-7, PMID: 39375543 PMC11659173

[20] Gannon J Bach K Cattaruzza MS Bar-Zeev Y Forberger S Kilibarda B. Big tobacco's dirty tricks: seven key tactics of the tobacco industry. Tob Prev Cessat. (2023) 9:1–9. doi: 10.18332/tpc/176336, PMID: 38124801 PMC10731746

[21] Watts C Rose S McGill B Yazidjoglou A. New image, same tactics: global tobacco and vaping industry strategies to promote youth vaping. Health Promot Int. (2024) 39:126. doi: 10.1093/heapro/daae126, PMID: 39495009 PMC11533144

[22] World Health Organization. (2024). The Global Health Observatory: Age-standardized estimates of current tobacco use, tobacco smoking and cigarette smoking (Tobacco control: Monitor). Available online at: https://www.who.int/data/gho/data/indicators/indicator-details/GHO/gho-tobacco-control-monitor-current-tobaccouse-tobaccosmoking-cigarrettesmoking-agestd-tobagestdcurr (Accessed April 21, 2025).

[23] The WHO Regional Office for the Eastern Mediterranean (2025) Religion and tobacco use. Available online at: https://www.emro.who.int/tfi/ban-tobacco/religion-and-tobacco-use.html (Accessed April 21, 2025).

[24] Baroud S Eladl M Aboelkheir A Mahmoud I. Knowledge, practices and reasons of hookah smoking in the United Arab Emirates: a cross-sectional study. Hamdan Med J. (2021) 14:17–22. doi: 10.4103/HMJ.HMJ_63_20

[25] Maziak W Nakkash R Bahelah R Husseini A Fanous N Eissenberg T. Tobacco in the Arab world: old and new epidemics amidst policy paralysis. Health Policy Plan. (2014) 29:784–94. doi: 10.1093/heapol/czt055, PMID: 23958628 PMC4153301

[26] Nasser AMA Geng Y Al-Wesabi SA. The prevalence of smoking (cigarette and Waterpipe) among university students in some Arab countries: a systematic review. Asian Pac J Cancer Prev. (2020) 21:583–91. doi: 10.31557/apjcp.2020.21.3.583, PMID: 32212782 PMC7437327

[27] Salloum RG Lee J Mostafa A Abu-Rmeileh NME Hamadeh RR Darawad MW. Waterpipe Tobacco smoking among university students in three eastern Mediterranean countries: patterns, place, and Price. Subst Use Misuse. (2019) 54:2275–83. doi: 10.1080/10826084.2019.1645177, PMID: 31347433

[28] Abbasi Y Hout MV Faragalla M Itani L. Knowledge and use of electronic cigarettes in young adults in the United Arab Emirates, particularly during the COVID-19 pandemic. Int J Environ Res Public Health. (2022) 19:7828. doi: 10.3390/ijerph19137828, PMID: 35805487 PMC9265798

[29] Ahmed LA Verlinden M Alobeidli MA Alahbabi RH AlKatheeri R Saddik B. Patterns of Tobacco smoking and nicotine vaping among university students in the United Arab Emirates: a cross-sectional study. Int J Environ Res Public Health. (2021) 18:7652. doi: 10.3390/ijerph18147652, PMID: 34300103 PMC8306162

[30] Kheirallah KA Alsulaiman JW Mohammad HA Veeranki SP Ward KD. Waterpipe tobacco smoking among Arab youth; a cross-country study. Ethn Dis. (2016) 26:107–12. doi: 10.18865/ed.26.1.107 26843803 PMC4738847

[31] Rababah JA Al-Hammouri MM. Health literacy and smoking habits among a sample of Jordanian university students. J Community Health. (2023) 48:30–7. doi: 10.1007/s10900-022-01139-8, PMID: 36107378

[32] Jawad M Abdulrahim S Daouk A. The social patterning of Tobacco use among women in Jordan: the protective effect of education on cigarette smoking and the deleterious effect of wealth on cigarette and Waterpipe smoking. Nicotine Tob Res. (2016) 18:379–85. doi: 10.1093/ntr/ntv111, PMID: 26014452

[33] Khattab A Javaid A Iraqi G Alzaabi A Ben Kheder A Koniski ML. Smoking habits in the Middle East and North Africa: results of the BREATHE study. Respir Med. (2012) 106:S16–24. doi: 10.1016/s0954-6111(12)70011-2 23290700

[34] Al-Hamdani M Manly E. Smoking cessation or initiation: the paradox of vaping. Prev Med Rep. (2021) 22:101363. doi: 10.1016/j.pmedr.2021.101363, PMID: 33868902 PMC8044675

[35] McKee M Capewell S. Evidence about electronic cigarettes: a foundation built on rock or sand? BMJ. (2015) 351:h4863. doi: 10.1136/bmj.h4863, PMID: 26374616

[36] Hersi M Beck A Hamel C Esmaeilisaraji L Pussegoda K Austin B. Effectiveness of smoking cessation interventions among adults: an overview of systematic reviews. Syst Rev. (2024) 13:179. doi: 10.1186/s13643-024-02570-9, PMID: 38997788 PMC11242003

[37] Lindson-Hawley N Hartmann-Boyce J Fanshawe TR Begh R Farley A Lancaster T. Interventions to reduce harm from continued tobacco use. Cochrane Database Syst Rev. (2016) 10:Cd005231. doi: 10.1002/14651858.CD005231.pub3, PMID: 27734465 PMC6463938

[38] Collins L Glasser AM Abudayyeh H Pearson JL Villanti AC. E-cigarette marketing and communication: how E-cigarette companies market E-cigarettes and the public engages with E-cigarette information. Nicotine Tob Res. (2019) 21:14–24. doi: 10.1093/ntr/ntx284, PMID: 29315420 PMC6610165

[39] Ma H Noar SM Ribisl KM. Associations of e-cigarette advertising exposure with curiosity and susceptibility among U.S. adolescents: national youth tobacco surveys, 2014-2020. PLoS One. (2024) 19:e0303903. doi: 10.1371/journal.pone.0303903, PMID: 39302930 PMC11414972

[40] Ranjit A McCutchan G Brain K Poole R. “That’s the whole thing about vaping, it’s custom tasty goodness”: a meta-ethnography of young adults’ perceptions and experiences of e-cigarette use. Subst Abuse Treat Prev Policy. (2021) 16:85. doi: 10.1186/s13011-021-00416-4, PMID: 34772440 PMC8586839

[41] Vogel EA Ramo DE Rubinstein ML Delucchi KL Darrow SM Costello C. Effects of social media on adolescents' willingness and intention to use E-cigarettes: an experimental investigation. Nicotine Tob Res. (2021) 23:694–701. doi: 10.1093/ntr/ntaa003, PMID: 31912147 PMC7976937

[42] Bandi P Star J Minihan AK Patel M Nargis N Jemal A. Changes in E-cigarette use among U.S. adults, 2019-2021. Am J Prev Med. (2023) 65:322–6. doi: 10.1016/j.amepre.2023.02.026, PMID: 37479423

[43] Fadus MC Smith TT Squeglia LM. The rise of e-cigarettes, pod mod devices, and JUUL among youth: factors influencing use, health implications, and downstream effects. Drug Alcohol Depend. (2019) 201:85–93. doi: 10.1016/j.drugalcdep.2019.04.011, PMID: 31200279 PMC7183384

[44] Lyzwinski LN Naslund JA Miller CJ Eisenberg MJ. Global youth vaping and respiratory health: epidemiology, interventions, and policies. NPJ Prim Care Respir Med. (2022) 32:14. doi: 10.1038/s41533-022-00277-9, PMID: 35410990 PMC9001701

[45] Tattan-Birch H Brown J Shahab L Beard E Jackson SE. Trends in vaping and smoking following the rise of disposable e-cigarettes: a repeat cross-sectional study in England between 2016 and 2023. Lancet Reg Health Eur. (2024) 42:100924. doi: 10.1016/j.lanepe.2024.100924, PMID: 39070753 PMC11281926

[46] Awad AA Itumalla R Gaidhane AM Khatib MN Ballal S Bansal P. Association of electronic cigarette use and suicidal behaviors: a systematic review and meta-analysis. BMC Psychiatry. (2024) 24:608. doi: 10.1186/s12888-024-06012-7, PMID: 39256668 PMC11389297

[47] Neczypor EW Mears MJ Ghosh A Sassano MF Gumina RJ Wold LE. E-cigarettes and cardiopulmonary health: review for clinicians. Circulation. (2022) 145:219–32. doi: 10.1161/circulationaha.121.056777, PMID: 35041473 PMC8820458

[48] Osei AD Mirbolouk M Orimoloye OA Dzaye O Uddin SMI Benjamin EJ. Association between E-cigarette use and chronic obstructive pulmonary disease by smoking status: behavioral risk factor surveillance system 2016 and 2017. Am J Prev Med. (2020) 58:336–42. doi: 10.1016/j.amepre.2019.10.014, PMID: 31902685 PMC9843649

[49] Shabil M Khatib MN Ballal S Bansal P Tomar BS Ashraf A. The impact of electronic cigarette use on periodontitis and periodontal outcomes: a systematic review and meta-analysis. BMC Oral Health. (2024) 24:1197. doi: 10.1186/s12903-024-05018-7, PMID: 39385155 PMC11463078

[50] Xie W Kathuria H Galiatsatos P Blaha MJ Hamburg NM Robertson RM. Association of Electronic Cigarette use with Incident Respiratory Conditions among US adults from 2013 to 2018. JAMA Netw Open. (2020) 3:e2020816. doi: 10.1001/jamanetworkopen.2020.20816, PMID: 33180127 PMC7662143

[51] Grundinger N Andreas M Lohner V Schneider S Mons U Vollstädt-Klein S. From smoking to vaping: the motivation for E-cigarette use at the neurobiological level-an fMRI study. Nicotine Tob Res. (2024) 27:1236–46. doi: 10.1093/ntr/ntae273 PMC12187334 39569465

[52] Poole R Carver H Anagnostou D Edwards A Moore G Smith P. Tobacco use, smoking identities and pathways into and out of smoking among young adults: a meta-ethnography. Subst Abuse Treat Prev Policy. (2022) 17:24. doi: 10.1186/s13011-022-00451-9, PMID: 35346260 PMC8960094

[53] Tiwari RK Sharma V Pandey RK Shukla SS. Nicotine addiction: neurobiology and mechanism. J Pharmacopuncture. (2020) 23:1–7. doi: 10.3831/kpi.2020.23.001, PMID: 32322429 PMC7163392

[54] Al-Sawalha NA Almomani BA Mokhemer E Al-Shatnawi SF Bdeir R. E-cigarettes use among university students in Jordan: perception and related knowledge. PLoS One. (2021) 16:e0262090. doi: 10.1371/journal.pone.0262090, PMID: 34972196 PMC8719738

[55] Aldhahir AM Siraj RA Alqarni AA Alqahtani JS Alyami MM Majrshi MS. The prevalence and sociodemographic determinants of tobacco and nicotine use among students in healthcare disciplines in Saudi Arabian universities: a cross-sectional survey. Front Public Health. (2024) 12:1348370. doi: 10.3389/fpubh.2024.1348370, PMID: 38515594 PMC10954892

[56] Alduraywish SA Aldakheel FM Alsuhaibani OS Jabaan ADB Alballa RS Alrashed AW. Knowledge and attitude toward e-cigarettes among first year university students in Riyadh, Saudi Arabia. Healthcare. (2023) 11:502. doi: 10.3390/healthcare11040502, PMID: 36833037 PMC9957237

[57] AlSayyad AS Alajaimi BA Matar E Abdulla SI Alaradi FA Salman MAA. Gender differences in e-cigarette knowledge, attitudes, and practice among adults in Bahrain: a cross-sectional study. Discover Public Health. (2024) 21:116. doi: 10.1186/s12982-024-00237-3

[58] Mostafa OA Taha MA. Knowledge, attitude, and use of electronic cigarettes among Cairo University medical students. J Egypt Public Health Assoc. (2024) 99:29. doi: 10.1186/s42506-024-00177-5, PMID: 39586909 PMC11589016

[59] Nazzal Z Maraqa B Azizeh R Darawsha B AbuAlrub I Hmeidat M. Exploring the prevalence, knowledge, attitudes and influencing factors of e-cigarette use among university students in Palestine: a cross-sectional study. BMJ Open. (2024) 14:e080881. doi: 10.1136/bmjopen-2023-080881, PMID: 38367977 PMC10875484

[60] Blackham-Hayward E Kertesz Z Chichger H. Electronic vape fluid activates the pulmonary endothelium and disrupts vascular integrity in vitro through an ARF6-dependent pathway. Microvasc Res. (2024) 153:104653. doi: 10.1016/j.mvr.2024.104653, PMID: 38220030

[61] Chatterjee S Tao JQ Johncola A Guo W Caporale A Langham MC. Acute exposure to e-cigarettes causes inflammation and pulmonary endothelial oxidative stress in nonsmoking, healthy young subjects. Am J Physiol Lung Cell Mol Physiol. (2019) 317:L155–l166. doi: 10.1152/ajplung.00110.2019, PMID: 31042077 PMC6734380

[62] Espinoza-Derout J Shao XM Lao CJ Hasan KM Rivera JC Jordan MC. Electronic cigarette use and the risk of cardiovascular diseases. Front Cardiovasc Med. (2022) 9:879726. doi: 10.3389/fcvm.2022.879726, PMID: 35463745 PMC9021536

[63] Kundu A Sachdeva K Feore A Sanchez S Sutton M Seth S. Evidence update on the cancer risk of vaping e-cigarettes: a systematic review. Tob Induc Dis. (2025) 23:1–14. doi: 10.18332/tid/192934, PMID: 39877383 PMC11773639

[64] Lee J Yao Z Boakye E Blaha MJ. The impact of chronic electronic cigarette use on endothelial dysfunction measured by flow-mediated vasodilation: a systematic review and meta-analysis. Tob Induc Dis. (2024) 22:1–10. doi: 10.18332/tid/186932, PMID: 38779295 PMC11110651

[65] Baldassarri SR Fiellin DA Friedman AS. Vaping-seeking clarity in a time of uncertainty. JAMA. (2019) 322:1951–2. doi: 10.1001/jama.2019.16493, PMID: 31697311 PMC8284981

[66] Izquierdo-Condoy JS Naranjo-Lara P Morales-Lapo E Hidalgo MR Tello-de-la-Torre A Vásconez-Gonzáles E. Direct health implications of e-cigarette use: a systematic scoping review with evidence assessment. Front Public Health. (2024) 12:1427752. doi: 10.3389/fpubh.2024.1427752, PMID: 39135931 PMC11317248

[67] Sobczak A Kośmider L Koszowski B Goniewicz ML. E-cigarettes and their impact on health: from pharmacology to clinical implications. Pol Arch Intern Med. (2020) 130:668–75. doi: 10.20452/pamw.15229, PMID: 32155137 PMC7685201

[68] Reiter A Hébert-Losier A Mylocopos G Filion KB Windle SB O'Loughlin JL. Regulatory strategies for preventing and reducing nicotine vaping among youth: a systematic review. Am J Prev Med. (2024) 66:169–81. doi: 10.1016/j.amepre.2023.08.002, PMID: 37553038

[69] Jeong M Weiger C Uriarte C Wackowski OA Delnevo CD. Youth attention, perceptions, and appeal in response to e-cigarette advertising features: a focus group study. Prev Med Rep. (2024) 44:102789. doi: 10.1016/j.pmedr.2024.102789, PMID: 38979482 PMC11228789

[70] Ajzen I. The theory of planned behavior. Organ Behav Hum Decis Process. (1991) 50:179–211. doi: 10.1016/0749-5978(91)90020-T

[71] Van Lange P Kruglanski A Higgins E. The theory of planned behavior SAGE Publications Ltd (2012).

[72] Doherty J Davison J McLaughlin M Giles M Dunwoody L McDowell C. Prevalence, knowledge and factors associated with e-cigarette use among parents of secondary school children. Public Health Pract. (2022) 4:100334. doi: 10.1016/j.puhip.2022.100334, PMID: 36389259 PMC9664552

[73] Motos-Sellés P Cortés-Tomás M-T Giménez-Costa J-A. Theory of planned behavior factors influencing E-cigarette use among adolescents: a systematic review. Curr Addict Rep. (2025) 12:7. doi: 10.1007/s40429-025-00612-3

[74] Simpson EEA Davison J Doherty J Dunwoody L McDowell C McLaughlin M. Employing the theory of planned behaviour to design an e-cigarette education resource for use in secondary schools. BMC Public Health. (2022) 22:276. doi: 10.1186/s12889-022-12674-3, PMID: 35144592 PMC8832682

[75] Barakat M Abuarab R Alkharabsheh B Budair N Fareed M Kharabsheh R. Exploratory validation of a survey instrument based on the theory of planned behavior to assess vaping attitude and perceptions. Int J Epidemiol Public Health Res. (2025) 6:1–9. doi: 10.61148/2836-2810/IJEPHR/140

[76] Regmi PR Waithaka E Paudyal A Simkhada P van Teijlingen E. Guide to the design and application of online questionnaire surveys. Nepal J Epidemiol. (2016) 6:640–4. doi: 10.3126/nje.v6i4.17258, PMID: 28804676 PMC5506389

[77] Stratton SJ. Population research: convenience sampling strategies. Prehosp Disaster Med. (2021) 36:373–4. doi: 10.1017/S1049023X21000649 34284835

[78] Johnson TP. Snowball sampling: introduction In: Wiley stats ref: Statistics reference online (2014)

[79] Sergeant E. (2025). Epitools Epidemiological Calculators. Ausvet. Available online at: https://epitools.ausvet.com.au/ [Accessed January 5, 2025).

[80] Althubaiti A. Sample size determination: a practical guide for health researchers. J Gen Fam Med. (2023) 24:72–8. doi: 10.1002/jgf2.600, PMID: 36909790 PMC10000262

[81] World Health Organization. (2020) Global adult Tobacco survey. Available online at: https://www.who.int/teams/noncommunicable-diseases/surveillance/systems-tools/global-adult-tobacco-survey (Accessed April 23, 2025).

[82] Taber KS. The use of Cronbach’s alpha when developing and reporting research instruments in science education. Res Sci Educ. (2018) 48:1273–96. doi: 10.1007/s11165-016-9602-2

[83] Jasp Team (2024) JASP (Version 0.19.0) [Computer software] Available online at: https://jasp-stats.org/ (accessed 16 July 2025).

[84] Brown LD Cai TT DasGupta A. Interval estimation for a binomial proportion. Stat Sci. (2001) 16:101–33. doi: 10.1214/ss/1009213286

[85] van der Schaaf A Xu C-J van Luijk P van’t Veld AA Langendijk JA Schilstra C. Multivariate modeling of complications with data driven variable selection: guarding against overfitting and effects of data set size. Radiother Oncol. (2012) 105:115–21. doi: 10.1016/j.radonc.2011.12.006, PMID: 22264894

[86] Kim JH. Multicollinearity and misleading statistical results. Korean J Anesthesiol. (2019) 72:558–69. doi: 10.4097/kja.19087, PMID: 31304696 PMC6900425

[87] Fowler CD Gipson CD Kleykamp BA Rupprecht LE Harrell PT Rees VW. Basic science and public policy: informed regulation for nicotine and Tobacco products. Nicotine Tob Res. (2018) 20:789–99. doi: 10.1093/ntr/ntx175, PMID: 29065200 PMC5991436

[88] Unger JB Cruz T Shakib S Mock J Shields A Baezconde-Garbanati L. Exploring the cultural context of tobacco use: a transdisciplinary framework. Nicotine Tob Res. (2003) 5:S101–17. doi: 10.1080/14622200310001625546, PMID: 14668090

[89] Nur AA Leibbrand C Curran SR Votruba-Drzal E Gibson-Davis C. Managing and minimizing online survey questionnaire fraud: lessons from the triple C project. Int J Soc Res Methodol. (2024) 27:613–9. doi: 10.1080/13645579.2023.2229651, PMID: 39494158 PMC11529704

[90] Al-Hamdani M Brett Hopkins D. E-cigarettes in the Middle East: the known, unknown, and what needs to be known next. Prev Med Rep. (2023) 31:102089. doi: 10.1016/j.pmedr.2022.102089, PMID: 36530454 PMC9747636

[91] Albadrani MS Tobaiqi MA Muaddi MA Eltahir HM Abdoh ES Aljohani AM. A global prevalence of electronic nicotine delivery systems (ENDS) use among students: a systematic review and meta-analysis of 4, 189, 145 subjects. BMC Public Health. (2024) 24:3311. doi: 10.1186/s12889-024-20858-2, PMID: 39604914 PMC11603929

[92] Ghanim M Rabayaa M Abuawad M Saeedi M Amer J. E-cigarette use among university students in Palestine: prevalence, knowledge, and determinant factors. PLoS One. (2024) 19:e0302946. doi: 10.1371/journal.pone.0302946, PMID: 38718008 PMC11078419

[93] Jirjees F Dallal Bashi YH Kharaba Z Ahmadi K Barakat M AlObaidi H. Public awareness, prevalence, and regulations for the sale of electronic cigarettes in Arab countries: a narrative review. Tob Induc Dis. (2023) 21:1–17. doi: 10.18332/tid/168435, PMID: 37901882 PMC10603825

[94] Trumbo CW. Influence of risk perception on attitudes and norms regarding electronic cigarettes. Risk Anal. (2018) 38:906–16. doi: 10.1111/risa.12918, PMID: 29023906

[95] Villanueva-Blasco VJ Belda-Ferri L Vázquez-Martínez A. A systematic review on risk factors and reasons for e-cigarette use in adolescents. Tob Induc Dis. (2025) 23:1–25. doi: 10.18332/tid/196679, PMID: 39822244 PMC11734163

[96] Vogel EA Henriksen L Schleicher NC Prochaska JJ. Young people's e-cigarette risk perceptions, policy attitudes, and past-month nicotine vaping in 30 U.S. cities. Drug Alcohol Depend. (2021) 229:109122. doi: 10.1016/j.drugalcdep.2021.109122, PMID: 34695673 PMC8671354

[97] Manzione LC Shan L Azagba S. Associations between risk perceptions and cigarette, E-cigarette, and dual-product use among Canadian adolescents. Tob Use Insights. (2020) 13:1179173x20903784. doi: 10.1177/1179173x20903784, PMID: 32180684 PMC7057405

[98] Vichayanrat T Chidchuangchai W Karawekpanyawong R Phienudomkitlert K Chongcharoenjai N Fungkiat N. E-cigarette use, perceived risks, attitudes, opinions of e-cigarette policies, and associated factors among Thai university students. Tob Induc Dis. (2024) 22:1–10. doi: 10.18332/tid/186536, PMID: 38737769 PMC11087886

[99] Park JA Crotty Alexander LE Christiani DC. Vaping and Lung Inflammation and Injury. Annu Rev Physiol. (2022) 84:611–29. doi: 10.1146/annurev-physiol-061121-040014, PMID: 34724436 PMC10228557

[100] Zong H Hu Z Li W Wang M Zhou Q Li X. Electronic cigarettes and cardiovascular disease: epidemiological and biological links. Pflügers Archiv Europ J Physiol. (2024) 476:875–88. doi: 10.1007/s00424-024-02925-0, PMID: 38376568 PMC11139732

[101] Al-Naimi A Al-Obaidli F Al-Rashdi R FAZ C Al-Hamdani M. Sociodemographic characteristics and vaping motives as potential correlates of early vaping initiation. Front Public Health. (2024) 12:1484252. doi: 10.3389/fpubh.2024.1484252, PMID: 39839423 PMC11747700

[102] Hadisuyatmana S Prayudha AKSL Indarwati R Efendi F. The correlation between masculinity and smoking behavior among adolescent in Surabaya. J Global Pharma Technol. (2020) 12:795–804.

[103] Kodriati N Pursell L Hayati EN. A scoping review of men, masculinities, and smoking behavior: the importance of settings. Glob Health Action. (2018) 11:1589763. doi: 10.1080/16549716.2019.1589763, PMID: 30963822 PMC6461072

[104] Ng N Weinehall L Ohman A. If I don't smoke, I'm not a real man'--Indonesian teenage boys' views about smoking. Health Educ Res. (2007) 22:794–804. doi: 10.1093/her/cyl104, PMID: 16987943

[105] Govender D Bhana PD. Smoking, swearing and strong muscles: becoming boys in the primary school. Int J Educ Res. (2023) 121:102225. doi: 10.1016/j.ijer.2023.102225

[106] Wills AG Carey G. Adolescent peer choice and cigarette smoking: evidence of active gene-environment correlation? Twin Res Hum Genet. (2013) 16:970–6. doi: 10.1017/thg.2013.51, PMID: 23924806 PMC3997114

[107] David J-C Fonte D Sutter-Dallay A-L Auriacombe M Serre F Rascle N. The stigma of smoking among women: a systematic review. Soc Sci Med. (2024) 340:116491. doi: 10.1016/j.socscimed.2023.116491, PMID: 38096599

[108] Davis DR Buta E Green B Krishnan-Sarin S. Sex differences in appeal, reward, and sensory experience of E-cigarette flavors among adults who smoke cigarettes. Prev Med. (2024) 185:108040. doi: 10.1016/j.ypmed.2024.108040, PMID: 38866212 PMC11323236

[109] Goldenson NI Leventhal AM Simpson KA Barrington-Trimis JL. A review of the use and appeal of flavored electronic cigarettes. Curr Addict Rep. (2019) 6:98–113. doi: 10.1007/s40429-019-00244-4, PMID: 31453046 PMC6709993

[110] Pang RD Goldenson NI Kirkpatrick M Barrington-Trimis JL Cho J Leventhal AM. Sex differences in the appeal of flavored e-cigarettes among young adult e-cigarette users. Psychol Addict Behav. (2020) 34:303–7. doi: 10.1037/adb0000548, PMID: 31961168 PMC7064389

[111] Erik HE Çoban T Özcebe LH. The relationship between gender and women's Tobacco use: an ecological analysis with country-level data. Thorac Res Pract. (2025) 26:61–8. doi: 10.4274/ThoracResPract.2024.24072, PMID: 39930838 PMC11796305

[112] Kaleta D Polanska K Wojtysiak P Szatko F. Involuntary smoking in adolescents, their awareness of its harmfulness, and attitudes towards smoking in the presence of non-smokers. Int J Environ Res Public Health. (2017) 14:1095. doi: 10.3390/ijerph14101095, PMID: 28934143 PMC5664596

[113] East KA Hitchman SC McNeill A Thrasher JF Hammond D. Social norms towards smoking and vaping and associations with product use among youth in England, Canada, and the US. Drug Alcohol Depend. (2019) 205:107635. doi: 10.1016/j.drugalcdep.2019.107635, PMID: 31765990 PMC6905149

[114] Katz SJ Erkinnen M Lindgren B Hatsukami D. Beliefs about E-cigarettes: a focus group study with college students. Am J Health Behav. (2019) 43:76–87. doi: 10.5993/ajhb.43.1.7, PMID: 30522568 PMC7015258

[115] Lim CCW Sun T Vu G Chan GCK Leung J. The underbelly of E-cigarette advertising: regulating online markets on social media platforms. Harm Reduct J. (2024) 21:105. doi: 10.1186/s12954-024-01027-5, PMID: 38811969 PMC11134850

[116] Rutherford BN Lim CCW Cheng B Sun T Vu GT Johnson B. Viral vaping: a systematic review and meta analysis of e-cigarette and Tobacco-related social media content and its influence on youth behaviours and attitudes. Addict Behav. (2023) 147:107828. doi: 10.1016/j.addbeh.2023.107828, PMID: 37591107

[117] DeCicca P Kenkel D Lovenheim MF. The economics of Tobacco regulation: a comprehensive review. J Econ Lit. (2022) 60:883–970. doi: 10.1257/jel.20201482, PMID: 37075070 PMC10072869

[118] Hamadeh RR Lee J Abu-Rmeileh NME Darawad M Mostafa A Kheirallah KA. Gender differences in waterpipe tobacco smoking among university students in four eastern Mediterranean countries. Tob Induc Dis. (2020) 18:100. doi: 10.18332/tid/129266, PMID: 33299390 PMC7720794

[119] Hopkins DB Al-Hamdani M. Young Canadian e-cigarette users and the COVID-19 pandemic: examining vaping behaviors by pandemic onset and gender. Front Public Health. (2020) 8:620748. doi: 10.3389/fpubh.2020.620748, PMID: 33585389 PMC7874134

[120] Jawad M Lee JT Millett C. Waterpipe Tobacco smoking prevalence and correlates in 25 eastern Mediterranean and eastern European countries: cross-sectional analysis of the global youth Tobacco survey. Nicotine Tob Res. (2016) 18:395–402. doi: 10.1093/ntr/ntv101, PMID: 25957438

[121] Sibai AM Iskandarani M Darzi A Nakkash R Saleh S Fares S. Cigarette smoking in a middle eastern country and its association with hospitalisation use: a nationwide cross-sectional study. BMJ Open. (2016) 6:e009881. doi: 10.1136/bmjopen-2015-009881, PMID: 27059466 PMC4838686

[122] Vassey J Galimov A Kennedy CJ Vogel EA Unger JB. Frequency of social media use and exposure to tobacco or nicotine-related content in association with E-cigarette use among youth: a cross-sectional and longitudinal survey analysis. Prev Med Rep. (2022) 30:102055. doi: 10.1016/j.pmedr.2022.102055, PMID: 36531097 PMC9747649

[123] Vassey J Unger JB. Should tobacco-related marketing on social media have stronger restrictions? Commentary. Subst Use Misuse. (2023) 58:1615–9. doi: 10.1080/10826084.2023.2223287, PMID: 37442760 PMC11335318

[124] Vassey J Vogel EA Unger JB Cho J Bae D Donaldson SI. Impact of Instagram and TikTok influencer marketing on perceptions of e-cigarettes and perceptions of influencers in young adults: a randomised survey-based experiment. Tob Control. (2025):tc-2024-059021. doi: 10.1136/tc-2024-059021, PMID: 39947698 PMC12343919

[125] Blank ML Hoek J. Navigating social interactions and constructing vaping social identities: a qualitative exploration with New Zealand young adults who smoke. Drug Alcohol Rev. (2023) 42:268–76. doi: 10.1111/dar.13542, PMID: 36065162 PMC10087447

[126] Davidson M Al-Hamdani M. An examination of the social perceptions and vaping preferences of young electronic nicotine delivery system users. Front Public Health. (2023) 11:1150368. doi: 10.3389/fpubh.2023.1150368, PMID: 37151590 PMC10162018

[127] Donaldson CD Stupplebeen DA Fecho CL Ta T Zhang X Williams RJ. Nicotine vaping for relaxation and coping: race/ethnicity differences and social connectedness mechanisms. Addict Behav. (2022) 132:107365. doi: 10.1016/j.addbeh.2022.107365, PMID: 35605411

[128] Jha V Kraguljac A. Assessing the social influences, self-esteem, and stress of high school students who vape. Yale J Biol Med. (2021) 94:95–106. PMID: 33795986 PMC7995953

[129] Lee DN Kim HM Stevens EM. Association of Vaping Reasons with stress, anxiety, and depression among young adults who currently vape. Subst Use Misuse. (2025) 60:188–94. doi: 10.1080/10826084.2024.2422949, PMID: 39482818 PMC11710971

[130] Chen-Sankey JC Kong G Choi K. Perceived ease of flavored e-cigarette use and e-cigarette use progression among youth never tobacco users. PLoS One. (2019) 14:e0212353. doi: 10.1371/journal.pone.0212353, PMID: 30811486 PMC6392261

[131] Springer AE Davis C Van Dusen D Grayless M Case KR Craft M. School socioeconomic disparities in e-cigarette susceptibility and use among Central Texas middle school students. Prev Med Rep. (2018) 11:105–8. doi: 10.1016/j.pmedr.2018.05.014, PMID: 30023161 PMC6047056

[132] Fotuhi O Fong GT Zanna MP Borland R Yong HH Cummings KM. Patterns of cognitive dissonance-reducing beliefs among smokers: a longitudinal analysis from the international Tobacco control (ITC) four country survey. Tob Control. (2013) 22:52–8. doi: 10.1136/tobaccocontrol-2011-050139, PMID: 22218426 PMC4009366

[133] Al-Hamdani M Hopkins DB Hardardottir A Davidson M. Perceptions and experiences of vaping among youth and young adult E-cigarette users: considering age, gender, and Tobacco use. J Adolesc Health. (2021) 68:787–93. doi: 10.1016/j.jadohealth.2020.08.004, PMID: 32943292

[134] McArthur J Stewart S Al-Hamdani M. Vaping frequency in young users: the role of gender and age among regular users. Subst Use Misuse. (2024) 59:1778–84. doi: 10.1080/10826084.2024.2374975, PMID: 39028136

[135] Kong G Laestadius L Vassey J Majmundar A Stroup AM Meissner HI. Tobacco promotion restriction policies on social media. Tob Control. (2024) 33:398–403. doi: 10.1136/tc-2022-057348, PMID: 36328589 PMC10154427

[136] Laestadius L Van Hoorn K Vassey J. Tobacco, nicotine and counter-marketing promotions using Instagram's branded content tool. Tob Control. (2025) 34:369–73. doi: 10.1136/tc-2023-058301, PMID: 38160057 PMC11214637

[137] Vassey J Kennedy CJ Herbert Chang HC Smith AS Unger JB. Scalable surveillance of E-cigarette products on Instagram and TikTok using computer vision. Nicotine Tob Res. (2024) 26:552–60. doi: 10.1093/ntr/ntad224, PMID: 37947283 PMC11033573

[138] Vogel EA Unger JB Vassey J Barrington-Trimis JL. Effects of a nicotine warning label and vaping cessation resources on young adults' perceptions of pro-vaping instagram influencer posts. Addict Behav. (2024) 149:107888. doi: 10.1016/j.addbeh.2023.107888, PMID: 37857044 PMC10841614

[139] Laestadius L Vassey J Kim M Ozga J Li D Stanton C. Themes in e-liquid concept names as a marketing tactic: evidence from premarket Tobacco product applications in the USA. Tob Control. (2024) 33:412–3. doi: 10.1136/tc-2022-057657, PMID: 36171148 PMC10043038

[140] Vassey J Allem JP Barker J Cruz TB Pang R Unger JB. E-cigarette use and promotion by social media influencers during videogame play on twitch. Tob Control. (2023) 32:526–7. doi: 10.1136/tobaccocontrol-2021-056828, PMID: 34625511 PMC9153388

[141] Al-Jayyousi GF Shraim M Hassan DA Al-Hamdani M Kurdi R Hamad NA. University students' and staff attitudes toward the implementation of a "tobacco-free" policy: a view from Qatar. Prev Med Rep. (2024) 38:102605. doi: 10.1016/j.pmedr.2024.102605, PMID: 38292026 PMC10826297

[142] Al-Hamdani M Manly E. Harm reduction in tobacco control: where do we draw the line? J Public Health Policy. (2022) 43:149–54. doi: 10.1057/s41271-021-00327-5, PMID: 34997211

[143] Abouzoor R Al-Hamdani M. Differences in vaping frequency and negative health effects experienced from vaping in a sample of vapers from three middle eastern countries. Heliyon. (2025) 11:e42657. doi: 10.1016/j.heliyon.2025.e42657, PMID: 40051858 PMC11883400

