# Supplementary material for: Vaping leads tobacco consumption among university students in Arab countries: a study of behavioral and psychosocial factors associated with smoking
Source: Front Public Health. 2025 Aug 6;13:1636757. doi: 10.3389/fpubh.2025.1636757 (PMC12364872; doi:10.3389/fpubh.2025.1636757)
Supplement: Supplementary file 1 [file Data_Sheet_1.ZIP › Supplementary_results.docx]

Supplementary Material

# Supplementary Results

**Details of EFA and CFA**

A total of 21 items were subjected to exploratory factor analysis (EFA) using IBM SPSS Statistics for Windows, Version 26.0. Armonk, NY: IBM Corp. To ensure unidirectional scoring where higher values reflect more favorable attitudes toward vaping, five negatively worded items were reverse-coded prior to analysis. These included items related to health risks, nicotine addiction, anti-smoking policy awareness, and confidence in quitting or resisting vaping. Sampling adequacy was confirmed by the Kaiser-Meyer-Olkin (KMO) measure, which was 0.796, indicating a satisfactory level of common variance. Bartlett’s Test of Sphericity was significant (χ^2^(210) = 2594.97, *p* < .001), supporting the factorability of the correlation matrix. EFA using principal component analysis (PCA) extraction and oblimin rotation revealed five components with eigenvalues greater than 1, together explaining 62.4% of the total variance. The variance explained by each factor was as follows: Factor 1 (26.6%), Factor 2 (14.8%), Factor 3 (8.6%), Factor 4 (7.0%), and Factor 5 (5.3%). The scree plot showed (**Figure S1**) a clear inflection at five components, supporting the retention of a five-factor solution for further interpretation.

**Figure S1. Scree plot of eigenvalues for principal component analysis**


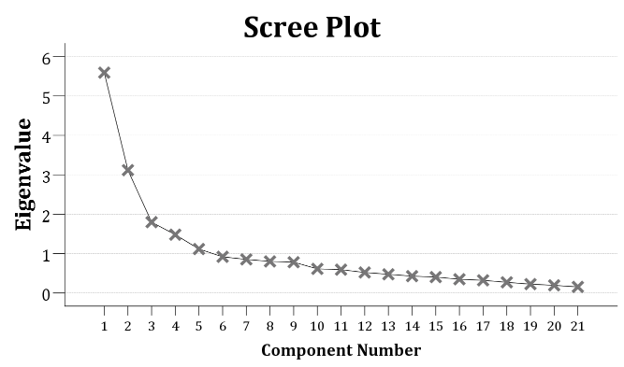


The final five-factor model was supported by both the pattern and structure matrices. After reverse coding five negatively worded items to ensure interpretive consistency, items loaded distinctly onto five interpretable components: harm reduction beliefs, health risk perceptions, self-efficacy, social and media influence, and economic accessibility. Most retained items showed primary loadings exceeding 0.60, indicating strong relationships with their respective factors. One item related to anti-smoking policy awareness was excluded due to weak loadings and lack of conceptual fit.

**Table S1. Component Matrix**

| **Item** | **Component** | | | | |
| --- | --- | --- | --- | --- | --- |
|  | 1 | 2 | 3 | 4 | 5 |
| I feel pressured to smoke by my peers | 0.465 | 0.493 |  |  |  |
| My role models in life consume tobacco in any form | 0.425 | 0.431 |  |  |  |
| Vaping is socially accepted among my peers | 0.462 |  |  |  |  |
| The portrayal of vaping in movies and series influence how I perceive it | 0.676 |  |  |  |  |
| The social media portrayal influence how I perceive vaping | 0.701 | 0.413 |  |  |  |
| I believe that vaping helps in social acceptance | 0.583 | 0.456 |  |  |  |
| I believe that vaping is less harmful compared to cigarettes | 0.679 |  |  | 0.481 |  |
| I believe that vaping is less harmful compared to Narghile | 0.584 |  |  | 0.481 |  |
| E-cigarettes (vaping) can help smokers quit smoking or cut down on smoking | 0.539 |  |  |  |  |
| I find vaping enjoyable | 0.688 |  |  |  |  |
| I find vaping satisfying | 0.721 |  |  |  |  |
| I believe that vaping is associated with health risks |  | 0.755 |  |  |  |
| I believe that vaping can lead to nicotine addiction |  | 0.699 |  |  |  |
| I am aware of anti-smoking policies within my university |  |  |  |  |  |
| Academic stress affects my vaping habits | 0.554 |  | 0.54 |  |  |
| I believe that vaping helps in stress reduction | 0.586 |  | 0.505 |  |  |
| I can easily obtain e-cigarettes | 0.455 | -0.49 |  |  |  |
| I believe that vaping is less costly compared to cigarettes | 0.54 |  |  |  | 0.623 |
| I believe that vaping is less costly compared to Narghile | 0.496 |  |  |  | 0.533 |
| I feel confident in my ability to resist vaping in social situations |  | 0.471 | 0.566 |  |  |
| I feel confident in my ability to quit vaping altogether |  |  | 0.573 |  | 0.44 |

Extraction Method: Principal Component Analysis; Five components extracted.

**Table S2. Pattern Matrix**

| Item | Component | | | | |
| --- | --- | --- | --- | --- | --- |
|  | Perceived Benefits | Behavioral Influence | Self-Efficacy | Social Influence | Economic |
| I feel pressured to smoke by my peers |  |  |  | -0.728 |  |
| My role models in life consume tobacco in any form |  |  |  | -0.752 |  |
| Vaping is socially accepted among my peers |  |  |  | -0.402 |  |
| The portrayal of vaping in movies and series influence how I perceive it |  |  |  | -0.83 |  |
| The social media portrayal influence how I perceive vaping |  |  |  | -0.776 |  |
| I believe that vaping helps in social acceptance |  |  |  | -0.722 |  |
| I believe that vaping is less harmful compared to cigarettes | 0.8 |  |  |  |  |
| I believe that vaping is less harmful compared to Narghile | 0.801 |  |  |  |  |
| E−cigarettes (vaping) can help smokers quit smoking or cut down on smoking | 0.668 |  |  |  |  |
| I find vaping enjoyable | 0.476 |  |  |  |  |
| I find vaping satisfying | 0.527 |  |  |  |  |
| I believe that vaping is associated with health risks |  | 0.754 |  |  |  |
| I believe that vaping can lead to nicotine addiction |  | 0.756 |  |  |  |
| I am aware of anti−smoking policies within my university |  |  |  |  |  |
| Academic stress affects my vaping habits |  | -0.614 |  |  |  |
| I believe that vaping helps in stress reduction | 0.426 | -0.624 |  |  |  |
| I can easily obtain e−cigarettes |  | -0.604 |  |  |  |
| I believe that vaping is less costly compared to cigarettes |  |  |  |  | 0.886 |
| I believe that vaping is less costly compared to Narghile |  |  |  |  | 0.848 |
| I feel confident in my ability to resist vaping in social situations |  |  | 0.67 |  |  |
| I feel confident in my ability to quit vaping altogether |  |  | 0.838 |  |  |

Extraction Method: Principal Component Analysis.

Rotation Method: Oblimin with Kaiser Normalization.

Rotation converged in 16 iterations.

**Table S3. Structure Matrix**

| Item | Component | | | | |
| --- | --- | --- | --- | --- | --- |
|  | 1 | 2 | 3 | 4 | 5 |
| I feel pressured to smoke by my peers |  |  |  | -0.715 |  |
| My role models in life consume tobacco in any form |  |  |  | -0.7 |  |
| Vaping is socially accepted among my peers |  | -0.432 |  | -0.401 |  |
| The portrayal of vaping in movies and series influence how I perceive it |  |  |  | -0.847 |  |
| The social media portrayal influence how I perceive vaping | 0.427 |  |  | -0.832 |  |
| I believe that vaping helps in social acceptance |  |  |  | -0.758 |  |
| I believe that vaping is less harmful compared to cigarettes | 0.833 |  |  |  | 0.419 |
| I believe that vaping is less harmful compared to Narghile | 0.781 |  |  |  |  |
| E-cigarettes (vaping) can help smokers quit smoking or cut down on smoking | 0.668 |  |  |  |  |
| I find vaping enjoyable | 0.625 |  |  | -0.517 |  |
| I find vaping satisfying | 0.674 |  |  | -0.478 |  |
| I believe that vaping is associated with health risks |  | 0.743 |  |  |  |
| I believe that vaping can lead to nicotine addiction |  | 0.727 |  |  |  |
| I am aware of anti-smoking policies within my university |  | 0.44 |  |  |  |
| Academic stress affects my vaping habits | 0.449 | -0.636 |  |  |  |
| I believe that vaping helps in stress reduction | 0.537 | -0.651 |  |  |  |
| I can easily obtain e-cigarettes |  | -0.665 |  |  |  |
| I believe that vaping is less costly compared to cigarettes |  |  |  |  | 0.887 |
| I believe that vaping is less costly compared to Narghile |  |  |  |  | 0.883 |
| I feel confident in my ability to resist vaping in social situations |  |  | 0.731 |  | -0.424 |
| I feel confident in my ability to quit vaping altogether |  |  | 0.822 |  |  |

Extraction Method: Principal Component Analysis.

Rotation Method: Oblimin with Kaiser Normalization.

Following exploratory analysis, the final CFA model in JASP software (Version 0.19.0) (Jasp Team, 2024), retained four latent constructs derived from prior EFA and grounded in the Theory of Planned Behavior (TPB): Perceived Benefits, Social Influence, Behavioral Influence - Risk, and Behavioral Influence - Situational Trigger. Items with standardized loadings below 0.50 were excluded to ensure adequate convergent validity, and subscales demonstrating low internal consistency (Cronbach’s α < 0.70) were removed. Specifically, the Self-Efficacy construct was excluded from the final model due to a Cronbach’s alpha below the 0.70 threshold, indicating insufficient internal reliability.

The final model demonstrated strong overall fit: χ^2^(48) = 122.16, *p* < .001; Comparative Fit Index (CFI) = 0.938; Tucker–Lewis Index (TLI) = 0.914; Root Mean Square Error of Approximation (RMSEA) = 0.074, with 90% confidence interval [0.058–0.090], *p* = .009; and Standardized Root Mean Square Residual (SRMR) = 0.054. These indices meet or exceed established thresholds for adequate model fit (e.g., CFI and TLI > 0.90; RMSEA and SRMR < 0.08), indicating that the hypothesized latent structure reliably accounts for the observed covariance among items. The RMSEA value remained below the conservative upper limit of 0.08, with a narrow confidence interval and a significant *p* value, reinforcing the precision and acceptability of the approximation.

Reliability analyses supported internal consistency across all four retained constructs. Coefficient omega (ω) values ranged from 0.782 to 0.807, and Cronbach’s alpha (α) values ranged from 0.771 to 0.799. The total scale demonstrated acceptable composite reliability (ω = 0.876).

**Table S4. Fit indices and reliability indicators of the modified VAPeS scale.**

| Model fit |  |  |  |
| --- | --- | --- | --- |
| Chi-square test |  |  |  |
| Model | χ^2^ | df | *p* |
| Baseline model | 1258.295 | 66 |  |
| Factor model | 122.16 | 48 | < .001 |
| Note.  The estimator is ML. |  |  |  |
| Additional fit measures |  |  |  |
| Fit indices |  |  |  |
| Index | Value |  |  |
| Comparative Fit Index (CFI) | 0.938 |  |  |
| Tucker-Lewis Index (TLI) | 0.914 |  |  |
| Bentler-Bonett Non-normed Fit Index (NNFI) | 0.914 |  |  |
| Bentler-Bonett Normed Fit Index (NFI) | 0.903 |  |  |
| Parsimony Normed Fit Index (PNFI) | 0.657 |  |  |
| Bollen's Relative Fit Index (RFI) | 0.867 |  |  |
| Bollen's Incremental Fit Index (IFI) | 0.939 |  |  |
| Relative Noncentrality Index (RNI) | 0.938 |  |  |
| Other fit measures |  |  |  |
| Metric | Value |  |  |
| Root mean square error of approximation (RMSEA) | 0.074 |  |  |
| RMSEA 90% CI lower bound | 0.058 |  |  |
| RMSEA 90% CI upper bound | 0.09 |  |  |
| RMSEA p-value | 0.009 |  |  |
| Standardized root mean square residual (SRMR) | 0.054 |  |  |
| Hoelter's critical N (α = .05) | 152.51 |  |  |
| Hoelter's critical N (α = .01) | 172.298 |  |  |
| Goodness of fit index (GFI) | 0.992 |  |  |
| McDonald fit index (MFI) | 0.878 |  |  |
| Expected cross validation index (ECVI) | 0.726 |  |  |
| Reliability |  |  |  |
| Social Influence | 0.784 | 0.787 |  |
| Perceived Benefits | 0.782 | 0.776 |  |
| Behavioral Influence - Risk | 0.804 | 0.799 |  |
| Behavioral Influence - Situational Trigger | 0.807 | 0.771 |  |
| total | 0.876 | 0.763 |  |

The final CFA model yielded a four-factor solution that aligned with theoretical expectations and demonstrated satisfactory convergent and discriminant validity. As illustrated in **Figure S2**, the latent constructs included Social Influence (ScI), Perceived Benefits (PrB), Behavioral Influence - Risk (BI-R), and Behavioral Influence- Situational Trigger (BI-ST). All observed indicators loaded significantly on their respective latent factors, with standardized loadings ranging from moderate (0.66) to high (0.97), indicating strong relationships between latent constructs and their observed variables. Notably, Social Influence exhibited the highest item loadings (0.72–0.91), reflecting a well-defined construct with robust internal structure. The Perceived Benefits factor also showed substantial item loadings (0.69–0.97), suggesting that beliefs about stress relief and accessibility meaningfully cluster together. Behavioral Influence – Risk and Situational Trigger demonstrated more moderate loadings, with BI-R items ranging from 0.66 to 0.78, and BI-ST items from 0.49 to 0.83. Residual variances were acceptable across all items, with no indicators showing excessive unexplained variance, supporting the reliability of observed variables. Inter-factor correlations provided evidence for construct discriminability: Perceived Benefits was moderately correlated with Social Influence (r = 0.38) and weakly with Behavioral Influence – Risk (r = 0.08), while Behavioral Influence – Risk was negatively correlated with Situational Triggers (r = –0.35), suggesting that students who vape due to situational cues (e.g., stress, availability) may not perceive risk as strongly motivating. These patterns support the conceptual distinction among the four constructs and reflect the multidimensional nature of vaping attitudes among university students.

**Figure S2. Confirmatory Factor Analysis (CFA) model for modified VAPeS scale.**


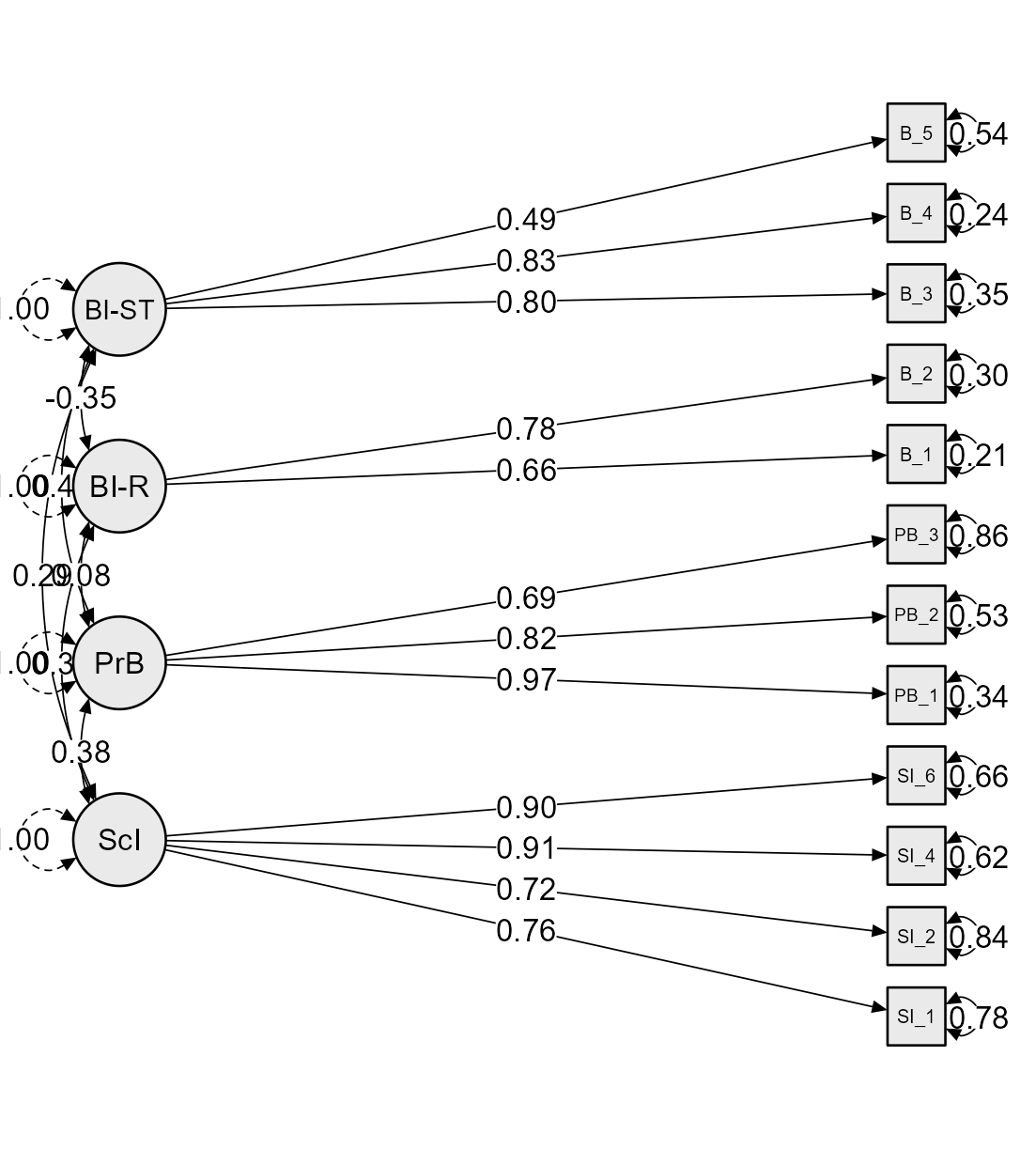


**References**

Jasp Team. (2024). *JASP (Version 0.19.0) [Computer software]*. In <https://jasp-stats.org/>
